# Methane–H_2_S Reforming Catalyzed by Carbon and Metal Sulfide Stabilized Sulfur Dimers

**DOI:** 10.1021/jacs.4c00738

**Published:** 2024-03-15

**Authors:** Yong Wang, Wenru Zhao, Xiaofeng Chen, Yinjie Ji, Xilei Zhu, Xiaomai Chen, Donghai Mei, Hui Shi, Johannes A. Lercher

**Affiliations:** † Department of Chemistry and Catalysis Research Center, Technische Universität München, Lichtenbergstrasse 4, 85748 Garching, Germany; ‡ School of Chemical Engineering and Technology, Tianjin University, Tianjin 300072, P. R. China; § School of Materials Science and Engineering, Tiangong University, Tianjin 300387, P. R. China; ∥ Institute for Integrated Catalysis, Pacific Northwest National Laboratory, P.O. Box 999, Richland, Washington 99352, United States; ⊥ School of Chemistry and Chemical Engineering, Yangzhou University, Yangzhou 225002, P. R. China

## Abstract

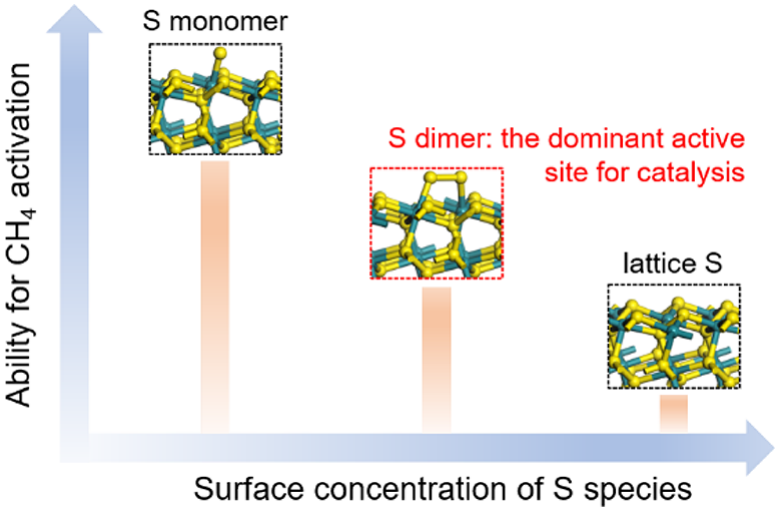

H_2_S reforming of methane (HRM) provides a potential strategy to directly utilize sour natural gas for the production of CO_*x*_-free H_2_ and sulfur chemicals. Several carbon allotropes were found to be active and selective for HRM, while the additional presence of transition metals led to further rate enhancements and outstanding stability (e.g., Ru supported on carbon black). Most metals are transformed to sulfides, but the carbon supports prevent sintering under the harsh reaction conditions. Supported by theoretical calculations, kinetic and isotopic investigations with representative catalysts showed that H_2_S decomposition and the recombination of surface H atoms are quasi-equilibrated, while the first C–H bond scission is the kinetically relevant step. Theory and experiments jointly establish that dynamically formed surface sulfur dimers are responsible for methane activation and catalytic turnovers on sulfide and carbon surfaces that are otherwise inert without reaction-derived active sites.

## Introduction

Methane is a key energy carrier in the transition to sustainable production of fuels and chemicals.^1−6^ Most approaches for methane utilization involve successive C–H bond cleavages to form surface carbons that are removed by co-reactant-derived oxygen species; these routes include steam and dry reforming (H_2_O or CO_2_ as co-reactant^7−15^), partial oxidation (O_2_ as co-reactant^16−24^), and autothermal reforming into CO_*x*_ and H_2_, the simplest building blocks that can be converted into a wide variety of carbon-containing products. Strategies have also been devised for direct selective C–H bond activation of methane into functionalized compounds that may be further upgraded (including halogenations,^25−30^ oxidative coupling,^31−39^ and partial oxidation^40−48^).

We recently investigated a route that uses H_2_S as a co-reactant for methane,^49^ H_2_S reforming of methane (HRM), which produces CS_2_ and H_2_. Compared to the other reforming options, this process has been less explored,^50−55^ not the least because of the hazardous nature of H_2_S, the unfavorable thermodynamics, and the high temperatures (>1000 K) needed for practically relevant conversions.

However, HRM not only enables the direct utilization of “sour” natural gas reserves that contain tremendous amounts of H_2_S but also provides a viable means to extract valuable hydrogen from H_2_S that otherwise goes into waste H_2_O in the Claus process.^56^ Preliminary techno-economic analyses indicated that HRM, when operated with carbon-neutral energy input, can be advantageous over steam methane reforming in terms of the H_2_ production cost.^57−59^ Moreover, when the potential process chain (Figure S1) is taken into account, the co-produced CS_2_ offers an entry point to a broad range of value-added sulfur chemicals and opens a nominally zero-CO_2_-emission path for the production of hydrocarbon fuels (e.g., CH_3_SH-to-hydrocarbons^60−62^).

Reforming with H_2_S occurs without a catalyst, but only at temperatures exceeding 900 °C (Figure S2), and suffers from parallel CH_4_ and H_2_S decomposition, leading to the formation of coke and sulfur residues, respectively.^63^ The relevance of these side reactions ultimately depends on a delicate balance of C* and S* (surface carbons and sulfurs) removal. Because H_2_S dissociation is fast and is not thermodynamically limited, higher and more stable rates of methane conversion require catalysts that not only activate C–H bonds efficiently but also remove C* via rapid C–S combination to vacate the surfaces for the next turnovers.

Recent studies on methane activation on metal catalysts showed that chemisorbed O*/OH* species on metal surfaces enable more efficient C–H bond activation than on bare surface metal atoms.^20−22,64,65^ Sulfur atoms may act similarly but to a milder degree.^34−36^ Therefore, it is intriguing to investigate the possibility of using sulfur atoms derived from H_2_S to assist in C–H bond activation, rather than O_2_ or H_2_O co-reactants.

Our previous study established several transition metal catalysts (in the form of oxides, oxysulfides, and sulfides) to be active and selective for HRM.^49^ However, these materials severely sintered under the reaction conditions, limiting their capabilities to disperse or host a high concentration of surface active sites. Thus, we decided to investigate high-surface-area materials that resist sintering at high temperatures (>900 K), such as carbons, as a support for the (pre)catalyst components.

We report in this work that carbon-based catalysts with high specific surface areas are active and selective for HRM. Even pure carbons exhibited considerable and relatively stable methane conversion rates for HRM. Substantial rate enhancements (by up to 5-fold) were achieved, however, by adding transition metals (mostly in sulfide forms under the reaction conditions). Through a combination of kinetic experiments, isotope labeling, and density-functional theory (DFT) calculations, key insights are achieved into the chemical identity of the active sites, the reaction mechanism, and the associated energetics. Most importantly, we show that dynamically formed surface sulfur dimers, but not monomers, catalyze methane activation and full catalytic turnovers on sulfide and carbon surfaces that are otherwise inert.

## Results and Discussion

### Catalytic HRM Activities on Select Carbon Materials with and without Metals

Before addressing the catalytic activities of carbon-supported catalysts, three carbon allotropes, carbon black (CB, specifically Vulcan XC72), carbon nanotubes, and graphene, were investigated. Surprisingly, these carbons turned out to be quite active for HRM (Figure S3). Carbon nanotubes were acid-washed, while the commercial graphene sample had been prepared by exfoliation in concentrated acids; trace metal impurities were removed by treatment of these carbon materials with concentrated HCl. The mass-specific rates tracked the specific surface areas (Figure S3), with the highest rate observed with graphene. The CB sample hardly deactivated further after losing ∼15% of the initial activity in the first 30 min of reaction, and structural changes of CB were not detected by Raman spectroscopy (Figure S4). Thus, it was chosen as the support for supporting the transition metals (M/CB catalysts) in subsequent experiments.

**Figure 1:**
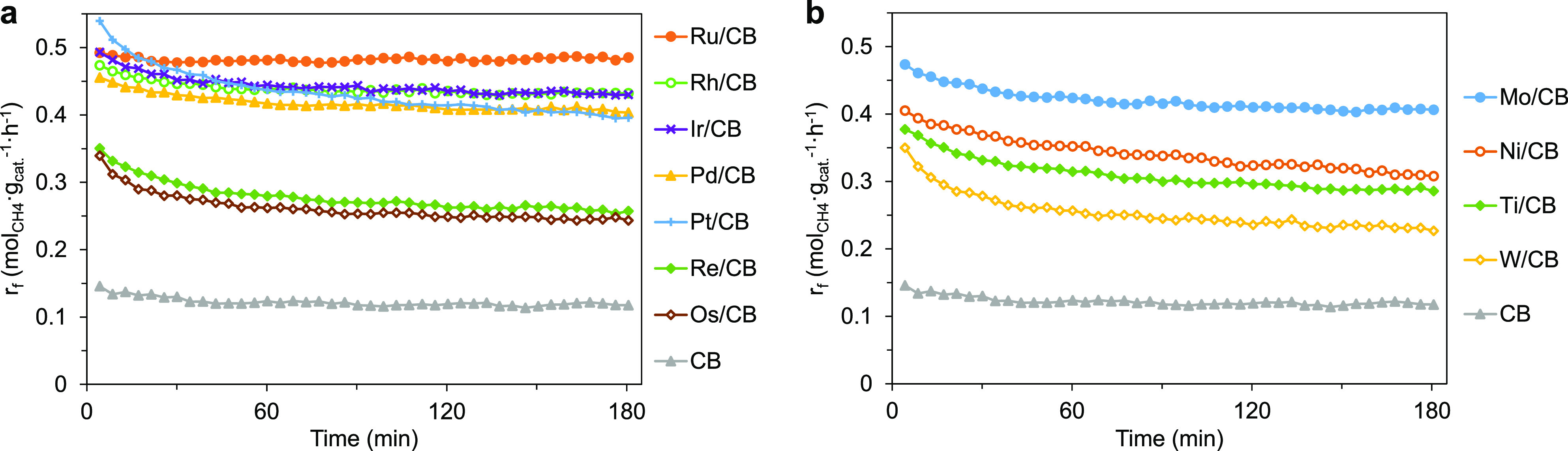
Catalytic stability of HRM over 5 wt % CB-supported (a) noble metals and (b) non-noble metals at 900 °C within a time-on-stream (TOS) of 3 h. Pretreatment conditions: 5 mg of catalyst, 20 mL/min of 10% H_2_S in H_2_ (1 bar), 900 °C, and 20 min. Reaction conditions: 0.08 bar CH_4_ and 0.24 bar H_2_S in He (1 bar), 48 L_CH_4__·g_cat._^–1^·h^–1^.

Seven noble metals (Ru, Rh, Pd, Re, Os, Ir, and Pt) and four non-noble metals (Ti, Ni, Mo, and W), supported on CB by wetness impregnation, were used as catalysts for HRM (Figure 1). Some of the elements (e.g., Ti, Mo, W) had been used in their bulk oxide forms (sulfided during reaction) in our previous work.^49^ Although metal components in these CB-supported (pre)catalysts still sintered under HRM conditions (XRD-determined crystallite sizes in Table S5), the mass-specific rates (after subtracting the contribution of CB itself) of Ti-, Mo-, and W-loaded CB catalysts were similar (0.10–0.28 mol_CH_4__·g_cat._^–1^·h^–1^) to those reported (0.10–0.20 mol_CH_4__·g_cat._^–1^·h^–1^) earlier for the bulk forms, despite an order of magnitude lower amount in the reactor. This suggests that the dispersion was about 1 order of magnitude higher compared to bulk samples.

Among the studied catalysts, 5 wt % Ru/CB was the most active on a mass basis (Figure 1). It was also exceptionally stable (Figure S5), while most other catalysts deactivated (Figure 1). Normalization to the estimated number of exposed metal sites in each 5 wt % M/CB catalyst (Table S5) shows that Pt/CB was the most active, having an apparent turnover frequency (TOF) of 12.4 s^–1^ at TOS of 3 h, i.e., 8-fold higher than the least active W/CB catalyst (apparent TOF: 1.5 s^–1^) under identical conditions. Although catalysts containing non-noble metals showed comparable mass-specific activities and are economically attractive, the most stable 5 wt % Ru/CB catalyst was selected, along with a reference catalyst (CB alone), for further kinetic and mechanistic investigations.

### Phase, Size, and Composition of CB-Supported Catalysts

**Figure 2:**
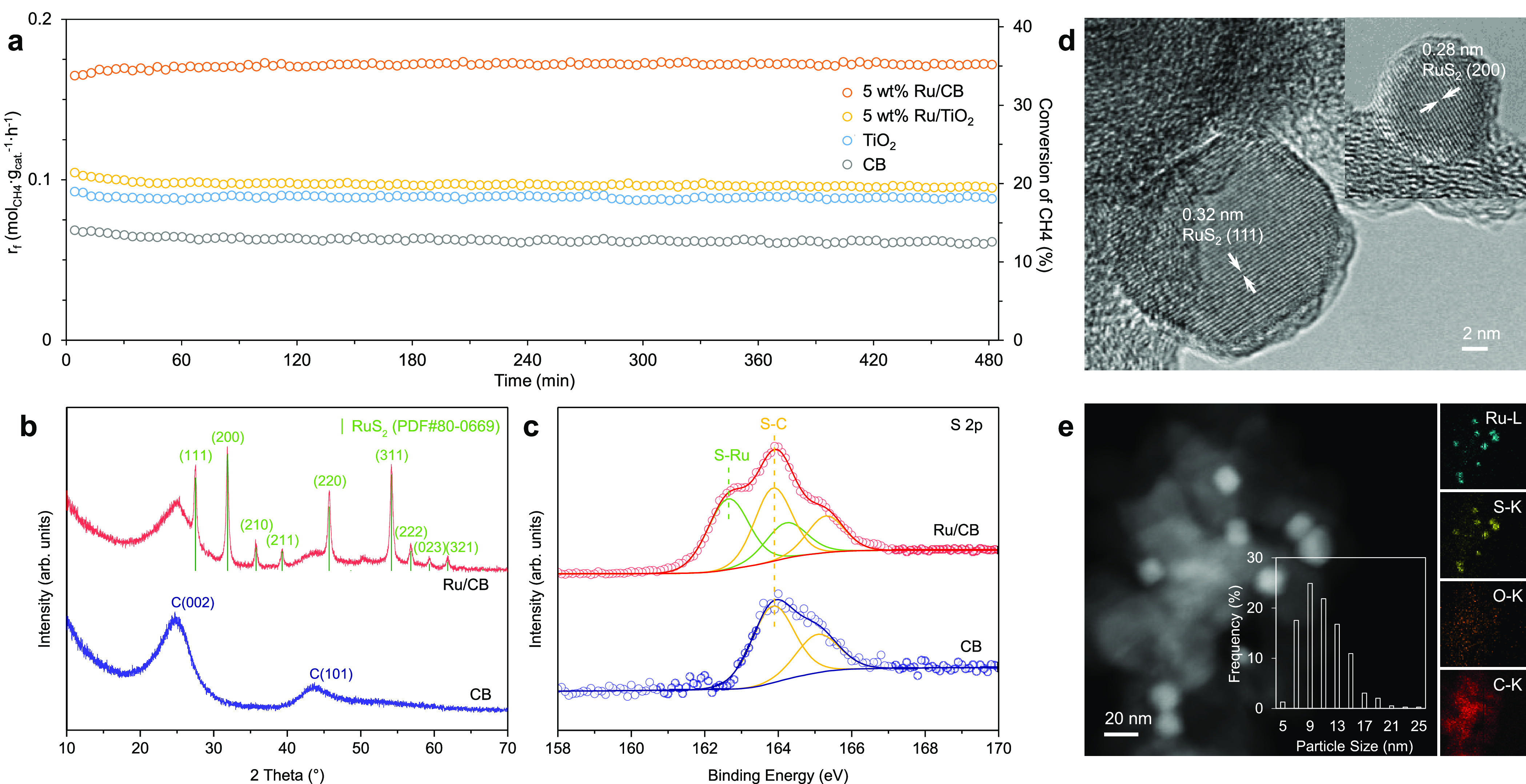
Catalytic performance of HRM over Ru/CB and CB catalysts and post-reaction structural characterizations. (a) Comparison of the catalytic performance between CB-supported and TiO_2_-supported Ru catalysts. Pretreatment conditions: 20 mg of catalyst diluted with 100 mg of quartz sand, 20 mL/min of 10% H_2_S in H_2_ (1 bar), 900 °C, 20 min. Reaction conditions: 0.08 bar CH_4_ and 0.24 bar H_2_S in He (1 bar), 12 L_CH_4__·g_cat._^–1^·h^–1^, and 900 °C. (b) XRD patterns of the spent Ru/CB and CB. (c) S 2p XPS spectra of the spent Ru/CB and CB. (d) High-resolution TEM images of the spent Ru/CB. (e) Dark-field TEM image of the spent Ru/CB and the energy-dispersive X-ray mapping images of Ru–L, S–K, O–K, and C–K.

In our previous study, nonstoichiometric Ti_2.45_S_4_ derived from the commercial P25 TiO_2_ showed an outstanding stability in HRM at 900 °C and, thus, was used as a benchmark in the present work. The catalytic performances of Ru/CB and CB are compared with those of the TiO_2_-based counterparts (Figure 2a). The mass-specific rate of 5 wt % Ru/CB was twice that of the TiO_2_-derived catalyst and three times that of CB, showing a compelling advantage that can be undoubtedly attributed to the loaded Ru. In contrast, loading 5 wt % Ru on TiO_2_ only marginally increased the activity compared to that of the TiO_2_-derived catalyst. Under HRM conditions, TiO_2_ was sulfided into Ti_2.45_S_4_ with a low surface area (4 m^2^/g),^49^ while Ru was converted to RuS_2_ (Figures 2b, S4b, and S5). For 5 wt % Ru/TiO_2_, the average crystallite size of RuS_2_ was 70 ± 20 nm according to the XRD analysis (Figure S6). In comparison, the average RuS_2_ crystallite size was much smaller in 5 wt % Ru/CB (Figure 2b; 18 ± 2 nm from XRD). This indicates that CB is superior to TiO_2_/TiS_*x*_ (which, as a support, undergoes phase transition and sintering) at dispersing the RuS_2_ domains.

High-resolution TEM images further confirmed the identity of nanoparticles on CB as RuS_2_ because the lattice spacings of 0.32 and 0.28 nm correspond to the (111) and (200) planes of the RuS_2_ phase, respectively (Figure 2d). The particle size distribution is based on a statistical analysis of ∼300 particles (Figure S7) and is shown as the inset of Figure 2e, illustrating that these RuS_2_ particles are mainly 5–20 nm in diameter. Figure 2e also shows the energy-dispersive X-ray mapping of a representative region, indicating the superimposed spatial distributions of S and Ru. The spent Ru/CB and CB were additionally characterized by ex situ XPS without exposing the samples to air during transfer. The S 2p doublet appears at 163.9 and 165.2 eV for the spent CB (Figure 2c), indicating the formation of the S–C bond on the carbon surface.^66^ For the spent Ru/CB, a new S 2p doublet appears at 162.6 and 164.2 eV, corresponding to the sulfidic S species in RuS_2_,^67^ and the ratio of S–Ru to S–C species is estimated to be 1:1. The Ru 3d_5/2_ peak is located at 280.2 eV in the spent Ru/CB sample (Figure S8), which is attributed to RuS_2_.^67^ These results indicate that the catalytic surface is in a sulfide state in Ru/CB.

For the other spent M/CB catalysts, all metals except Ir and Os were converted to the corresponding sulfide phases (Figure S9). The fact that the bulk phase of Ir- and Os-based catalysts remained metallic is attributed to the small equilibrium constants (*K*_eq_) of sulfidation for Ir and Os by H_2_S at 900 °C (i.e., ∼0.1, Table S6). Although the sulfidation *K*_eq_ is the largest for Ru among the investigated noble metals (∼66 at 900 °C, Table S6), the bulk phase in Ru/CB was still metallic Ru after pretreatment in 10% H_2_S/H_2_ at 900 °C for 20 min, while it was completely sulfided to RuS_2_ within 40 min at a higher ratio of *P*_H_2_S_ to *P*_H_2__ (∼3.7 on average along the catalyst bed) during the HRM reaction (Figure S10). The extent of bulk sulfidation did not significantly affect the activity of Ru/CB (Figure 2a), suggesting that the concentration of catalytically active sites or the exposed surfaces that host the active sites remained unchanged as the bulk phase was progressively sulfided. In addition, in a control experiment, the Ru/CB catalyst was pretreated in pure H_2_ to ensure that both the bulk phase and the surface of the Ru nanoparticles were in the metallic state; the initial catalytic activity was found to be identical to that of the fully sulfided Ru/CB (Figure S11), suggesting the same chemical identity of active sites (i.e., instantaneously formed S*, which will be discussed later) existed for both H_2_-reduced and sulfided catalysts.

### Kinetic and Isotopic Experiments on Representative Catalysts

**Figure 3:**
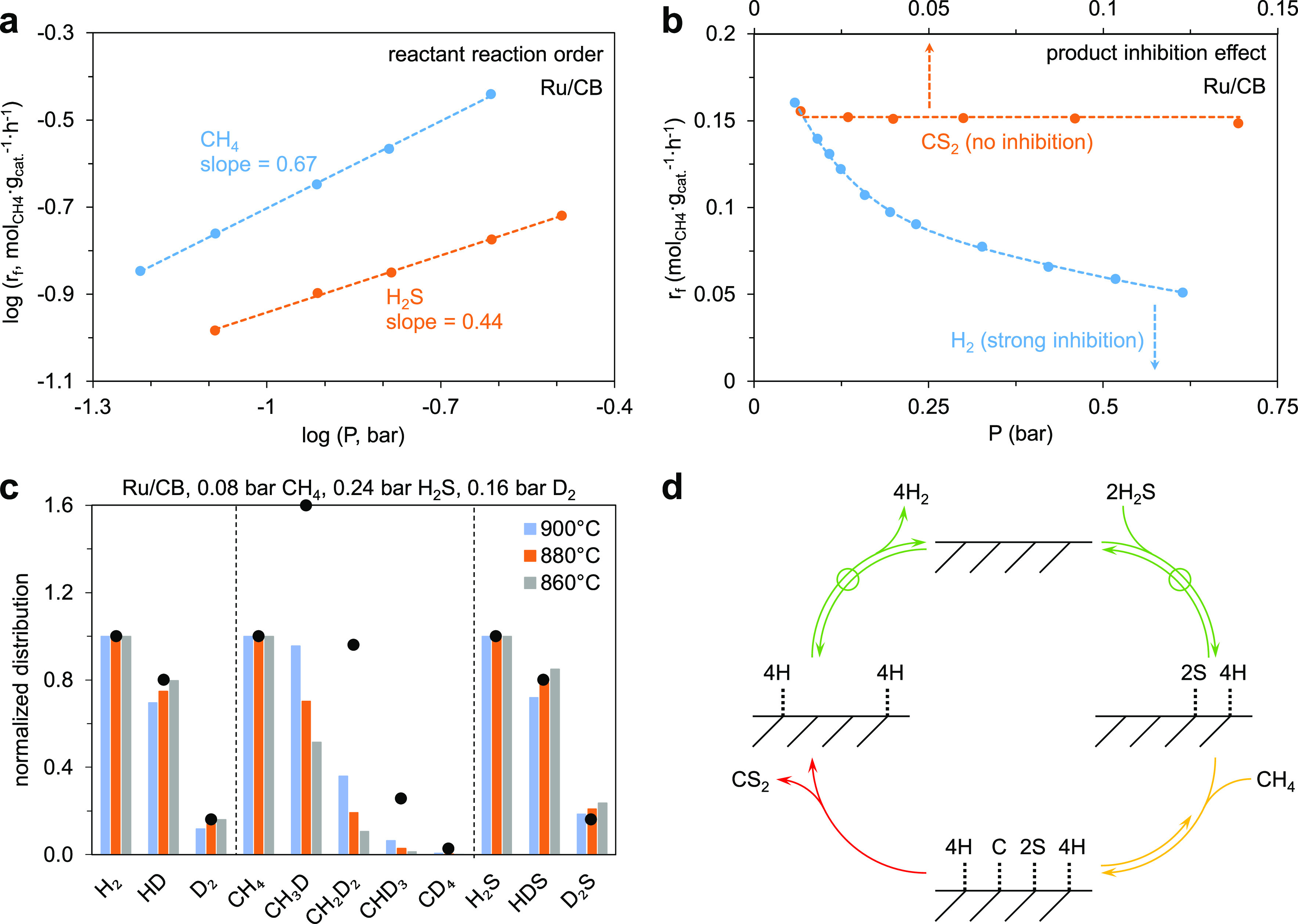
Kinetic and isotopic studies of HRM over Ru/CB. (a) Reaction orders of CH_4_ and H_2_S measured at a fixed partial pressure of 0.24 bar H_2_S (CH_4_ partial pressure varying between 0.06 and 0.24 bar) and 0.08 bar CH_4_ (H_2_S partial pressure varying between 0.08 and 0.24 bar), respectively, with a space velocity of 150 L·g_cat._^–1^·h^–1^, 900 °C. (b) Influence of co-feedings of H_2_ and CS_2_ on the forward rate of CH_4_ conversion. 0.08 bar CH_4_ and 0.24 bar H_2_S in He (1 bar), 12 L_CH_4__·g_cat._^–1^·h^–1^, 900 °C. (c) H/D isotopic exchange experiments over Ru/CB in a mixed flow of CH_4_, H_2_S, and D_2_ (12 L_CH_4__·g_cat._^–1^·h^–1^). The columns are the normalized isotopomer distributions of hydrogen (H_2_, HD, D_2_), methane (CH_4_, CH_3_D, CH_2_D_2_, CHD_3_, CD_4_), and hydrogen disulfide (H_2_S, HDS, D_2_S) detected by mass spectroscopy. The black dots are the binomial distribution for each molecule, assuming that all the decompositions of CH_4_, H_2_S, and D_2_ are reversible and then all the H and D can scramble in a statistical manner. (d) An illustrative representation of the catalytic HRM cycle over carbon-based catalysts obtained from the experimental study. Green lines indicate steps that are quasi-equilibrated; yellow lines indicate C–H bond scission steps that are reversible but not fully equilibrated (i.e., a lumped representation that does not specify the reversibility of the individual C–H scission steps); and red lines indicate the quasi-irreversible steps for the combination of C* and S* to form CS_2_.

To investigate the reaction mechanism of HRM on carbon-based catalysts, a series of kinetic and isotopic experiments were performed, mostly on two representative catalysts, 5 wt % Ru/CB and CB. The apparent reaction orders with respect to CH_4_ and H_2_S were fractional for both Ru/CB and CB (Figures 3a and S12a), pointing to significant surface coverages of species derived from both reactants. The reaction rate decreased substantially when co-feeding H_2_, while co-feeding CS_2_ did not change the reaction rate (Figures 3b and S12b). These results indicate that H_2_ dissociation and H* recombination are reversible, while CS_2_ formation via the combination of S* and C* or CS* is likely irreversible. At these temperatures, the H* coverages are expected to be negligible (the equilibrium constant for H_2_ dissociation was reported to be <10^–2^ bar^–1^ on metallic Ru and RuS_*x*_ surfaces even at lower temperatures such as 573–623 K^68−70^). Thus, the strong inhibitory effect of H_2_ should not reflect the competitive adsorption of H* with the reactive intermediates.

The H/D isotope exchange experiments were performed by co-feeding D_2_ together with CH_4_ and H_2_S to probe the reversibility of elementary steps that involve hydrogen atoms. As shown in Figures 3c and S13, the isotopomer distributions of dihydrogen (H_2_, HD, D_2_) and hydrogen sulfide (H_2_S, HDS, D_2_S) remained binomial across a wide range of reaction parameters (temperature, partial pressures of CH_4_, H_2_S, and D_2_, and space velocity) over Ru/CB. Note that the binomial distribution is calculated based on the total number of H and D in the system. For example, a mixture of 0.08 bar CH_4_, 0.24 bar H_2_S, and 0.16 bar D_2_ contains H and D atoms in a 5:2 ratio and full scrambling of these H and D atoms would lead to H_2_:HD:D_2_ and H_2_S:HDS:D_2_S ratios of 1.0:0.8:0.16, consistent with the measured values (1:0.75 ± 0.05:0.14 ± 0.02 for H_2_:HD:D_2_ and 1:0.79 ± 0.07:0.21 ± 0.03 for H_2_S:HDS:D_2_S in Figure 3c). In contrast, the isotopomer distribution of methane (CH_4_, CH_3_D, CH_2_D_2_, CHD_3_, and CD_4_) invariably deviated from the binomial distribution under all studied reaction conditions (black circles in Figures 3c and S13). The same patterns were observed for other carbon-supported catalysts (Pt/CB, Ir/CB) and CB alone (Figure S14).

These results indicate that the recombination of hydrogen adatoms (and its microscopic reverse, H_2_ dissociation) and H_2_S decomposition are quasi-equilibrated and that the CH_4_ decomposition steps are reversible but not quasi-equilibrated (Figure 3d). Interestingly, the C–H bond scission steps appear to be closer to equilibrium (binomial isotopomer distribution for CH_*x*_D_4–*x*_, *x* = 0–4) as the temperature and bed residence time increased (Figures S13 and S14).

**Figure 4:**
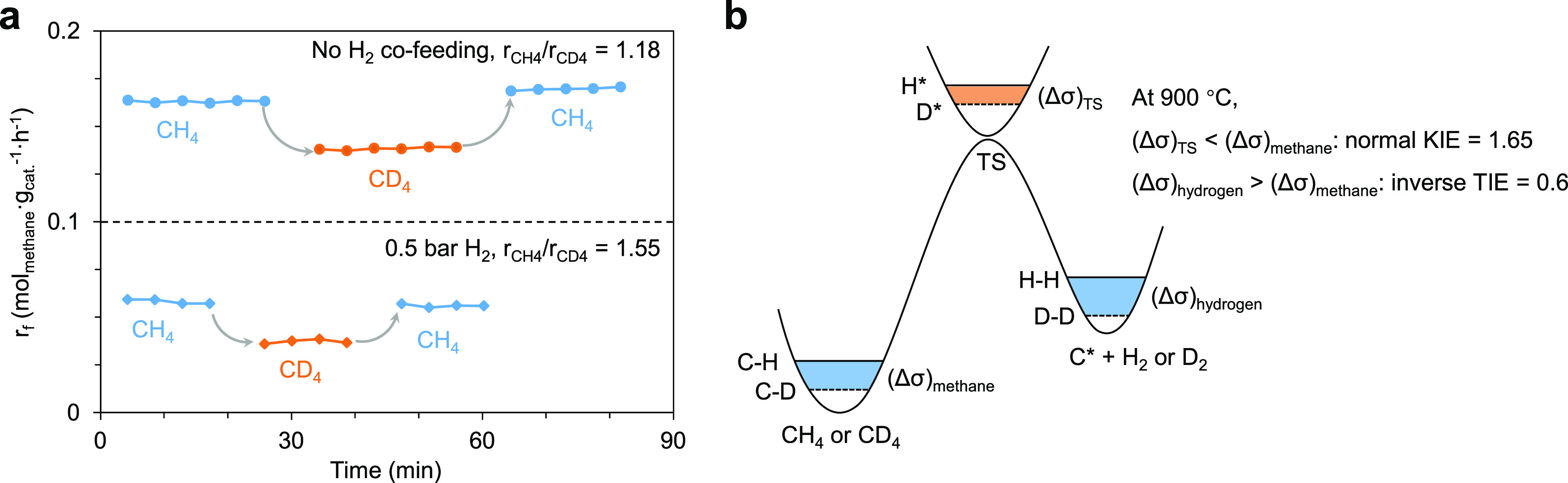
Kinetical relevance of C–H bond cleavage. (a) Kinetic isotope effect between CH_4_ and CD_4_ over Ru/CB. Reaction conditions: 0.08 bar of CH_4_/CD_4_ and 0.24 bar of H_2_S in He (1 bar), 12 L_CH_4__·g_cat._^–1^·h^–1^, and 900 °C. (b) Illustration of normal KIE and inverse TIE during the process of methane decomposition to hydrogen and C* on the catalyst surface.

The H/D kinetic isotope effect (KIE) was determined by measuring methane conversion rates with CH_4_–H_2_S and CD_4_–H_2_S reactant mixtures under steady-state conditions, and a normal KIE of ∼1.2 was observed for methane conversion rates in the absence of co-fed H_2_ on both Ru/CB (Figure 4a) and CB (Table S7). When varying the space velocity or co-feeding H_2_ up to 0.6 bar, the measured KIE varied in the range of 1.1–1.6 (Table S7), and closer inspection shows that KIE tends to increase at larger space velocities (shorter residence times) and higher partial pressures of co-fed H_2_. Because the C–H bond scission deviates more from equilibrium at shorter residence times and higher partial pressures of co-fed H_2_ (Figures S13 and S14), larger KIE values reflect a greater contribution from the zero-point energy (ZPE) difference between C–H and C–D (ΔZPE = 4.8 kJ mol^–1^, corresponding to a maximum KIE of 1.65 at 900 °C^71−77^). The decrease in KIE with increasing contact times or decreasing partial pressures of co-fed H_2_ reflects the increasing contribution of the inverse thermodynamic isotope effect (TIE) that originates from the methane decomposition (CH_4_ + * ⇌ C* + 2H_2_; CD_4_ + * ⇌ C* + 2D_2_) (Figure 4b). The theoretical isotope effect for CH_4_/CD_4_ decomposition (i.e., the ratio of the equilibrium constants for the two reactions) is calculated to be ∼0.6 at 900 °C. Our KIE data is consistent with the conclusion that recombinative H_2_ desorption and H_2_S dissociation steps do not limit the methane conversion rate and suggests instead that C–H bond cleavage is kinetically relevant.

For the activation of methane, the actual active site is hypothesized to be some form of dynamically formed surface sulfur species (designated as S* for the time being without implying its precise chemical structure) derived from quasi-equilibrated H_2_S decomposition. It should be emphasized that pure CB is inert for methane activation in the absence of H_2_S in agreement with experiment (Figure S15) and theory (Figure S16).

The fractional coverage of S* is determined by the equilibrium of H_2_S + * ⇌ H_2_ + S* (Figure 3d), where * is the host of the active site, presumably a certain type of C atom for pure CB and surface Ru cations for RuS_2_. With respect to the S*-assisted activation of C–H bonds in methane, there are two generic classes of reaction mechanisms that differ in the species (and its binding site) formed upon the first C–H bond scission, which is thought to form either H_3_C* (with the detached H bound to S*) or H_3_CS* (i.e., CH_3_ and S bind to the same *). These two fundamental types of mechanisms can be viewed as “competitive” and “non-competitive” mechanisms, respectively, with respect to whether C- and S-species are both bound to *. Within each category, there are subcases in which the reversibility of C–H dissociation steps may vary. The corresponding rate equations have been derived based on the proposed sequences of elementary steps. The detailed derivations can be found in Section 3 of the Supporting Information, where the involvement of lattice S in the catalytic cycle can be excluded based on the conflict between the measured rate data and the predicted trend (Situation III).

**Figure 5:**
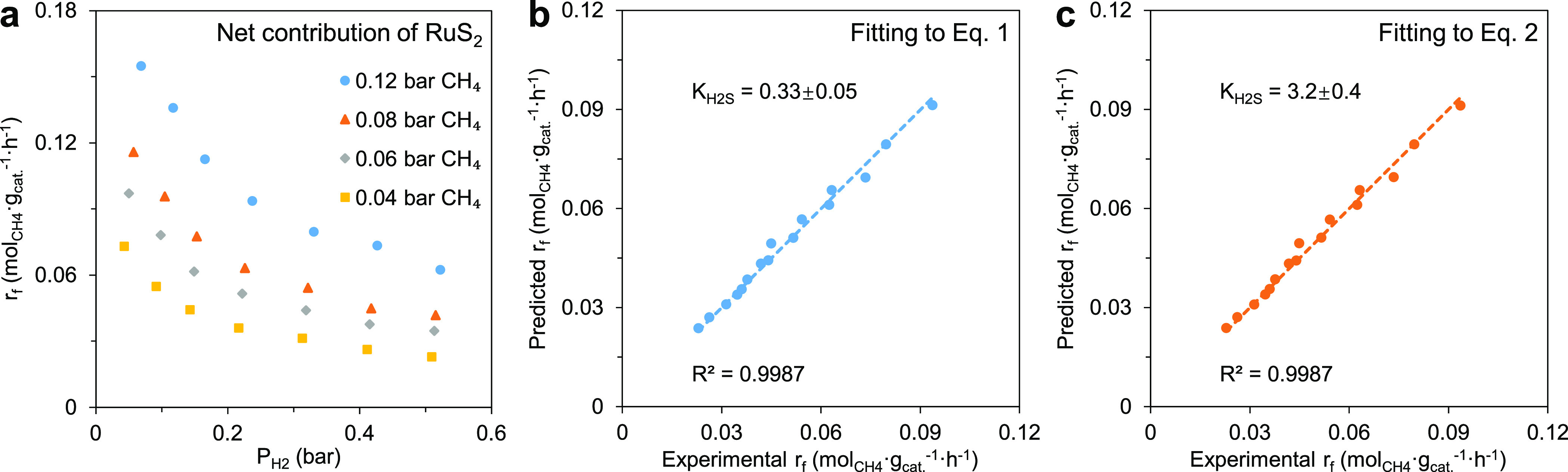
Experimental assessments of the competitive and non-competitive reaction mechanisms over RuS_2_ in the HRM reaction. (a) Net contribution of RuS_2_ to the methane conversion rate at 900 °C by subtracting the conversion rate over CB (Figure S17d) from that of Ru/CB (Figure S17a). (b, c) Parity plots of the predicted and measured methane conversion rates above 0.2 bar of H_2_ in (a). The predicted rates were obtained from fitting the measured rate data to eq 1 (b) and eq 2 (c), respectively, giving the regressed value of *n* between −0.1 and 0 for both but different *K*_H_2_S_ values with uncertainties representing the 95% confidence interval.

Through a series of experiments in which the partial pressures of reactants and co-fed H_2_ were varied in a wide range at several temperatures (860, 880, and 900 °C) with Ru/CB and CB (Figure S17), it was determined that the reaction order in CH_4_ reached unity when the co-fed H_2_ pressure was above 0.2 bar (Table 1, Figure S18, and Table S8). The first order in CH_4_ is a clear indication of low surface coverages of carbonaceous species (CH_x_*, *x* = 0–4). In this range of H_2_ pressure, for both “competitive” and “non-competitive” mechanisms (Situation I and II in Section 3 of the Supporting Information), the complex terms that contain CH_4_ and H_2_ pressure dependences can be approximated by a power form proportionate to [CH_4_]^1^ × [H_2_]^*n*^, regardless of the reversibility of C–H bond scissions. Thus, the rate equations can be simplified to the following formsSituation I, competitive mechanism:
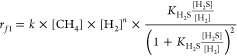
1Situation II, non-competitive mechanism:
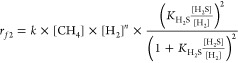
2where *k* is an apparent rate constant and *n* is an apparent reaction order in H_2_, both derived from regression to a power law formalism. The magnitude of *n* reflects the overall reversibility of sequential C–H bond scissions and the average composition of CH_x_*. Apparently, *n* must be always negative but less negative than the measured reaction order in H_2_ (>0.2 bar H_2_). In this range of H_2_ pressure (e.g., 0.2 bar), the measured reaction order in H_2_S was ∼0.60 for Ru/CB and ∼0.80 for CB (Table 1). A numerical analysis of eq 1 or 2, based on a series of simulated values of *K*_H_2_S_ and typical partial pressures of H_2_S and H_2_, showed that the functional dependence of [H_2_] associated solely with H_2_S decomposition equilibrium should be nearly identical to that of [H_2_S] in magnitude but with a negative sign. Comparing the measured reaction order in H_2_S with the measured reaction orders in H_2_ (−0.65 for Ru/CB and −0.80 for CB, Table 1), in turn, indicates that the overall H_2_ reaction order mainly stems from the H_2_S decomposition equilibrium, while the values of *n* contributed by the reversibility of methane decomposition are quite small. Using the rate data obtained above 0.2 bar H_2_ over Ru/CB and CB, which led to the first-order dependence on CH_4_ pressure, nonlinear least-squares fits to the universal rate expressions (parity plots and goodness of fits shown in Figures S21 and S22) yielded *n* values that are indeed small (between −0.1 and 0). While the goodness of fits is almost equally good for the two mechanisms, the regressed values of *K*_H_2_S_ are significantly different, i.e., approximately 0.3 (Ru/CB) and 0.1–0.2 (CB) for the competitive mechanism (Figure S21) and around 3 (Ru/CB) and 2 (CB) for the non-competitive mechanism (Figure S22). The regressed *K*_H_2_S_ values for Ru/CB (after subtracting the contribution from CB to the measured rate, Figure 5) are associated with the step H_2_S + * ⇌ H_2_ + S* which translates to Gibbs free energy changes (Δ*G*_rxn,900°C_^°^) of +10.8 and −11.3 kJ/mol (Table 2) for the competitive and non-competitive mechanisms, respectively.

**Table 1:** Reaction Orders in CH_4_, H_2_S, and H_2_ for HRM over Ru/CB and CB^a^

	CH_4_		H_2_S		H_2_	
Catalyst	No H_2_	>0.2 bar H_2_	No H_2_	0.2 bar H_2_	<0.20 bar	>0.2 bar
Ru/CB	0.66 ± 0.05	0.94 ± 0.08	0.50 ± 0.06	0.60 ± 0.05	–0.60 ± 0.10	–0.65 ± 0.10
CB	0.58 ± 0.15	0.97 ± 0.05	0.65 ± 0.05	0.80 ± 0.12	–0.70 ± 0.30	–0.80 ± 0.25

a See Figures 3a, S12, S18, S19, and S20 and the corresponding Tables S8 and S9 for the calculation of reaction orders.

**Table 2:** Comparison of Thermodynamic Parameters for H_2_S + * ⇌ H_2_ + S* (or H_2_S + ^1^/_2_*–* ⇌ H_2_ + ^1^/_2_S*–S*) Obtained from Experimental Data and Theoretical Calculations

Method	Equation or Model	*K*_H_2_S,900°C_	Δ*G*_rxn,900°C_^°^ (kJ/mol)	Δ*H*_rxn,900°C_^°^ (kJ/mol)
Experimental results^a^	Fitting to eq 1	0.33	+10.8	<0^b^
	Fitting to eq 2	3.2	–11.3	<0^b^
DFT calculations^c^	Forming S* monomer on RuS_2_(100)	0.09	+23.9	+2.8
	Forming S*–S* dimer on RuS_2_(100)	0.24	+14.0	–20.1
	Forming S*–S* dimer on RuS_2_(111)	0.39	+9.2	–35.2

a The *K*_H_2_S,900°C_ values were obtained from Figure 5, and the Δ*G*_rxn,900°C_^°^ values (per mole of S) were calculated by the equation Δ*G*° = −*RT* ln *K*.

b Δ*H*_rxn,900°C_^°^ should be a negative value, but the precise value cannot be determined due to the large uncertainties in the measured *K*_H_2_S_ values at lower temperatures (see the discussion below Figure S21).

c Δ*G*_rxn,900°C_^°^ and Δ*H*_rxn,900°C_^°^ were calculated through the correction of entropy at 900 °C for all the atoms involved in the reaction, including the surface Ru and S atoms. To ensure the accuracy of the DFT calculation, these values for the gas-phase reactions of the main HRM reaction and H_2_S decomposition were calculated by the same method, which are quite consistent with the results calculated from the HSC Chemistry database (Table S10).

### Theoretical Assessments of the Reaction Mechanism

**Figure 6:**
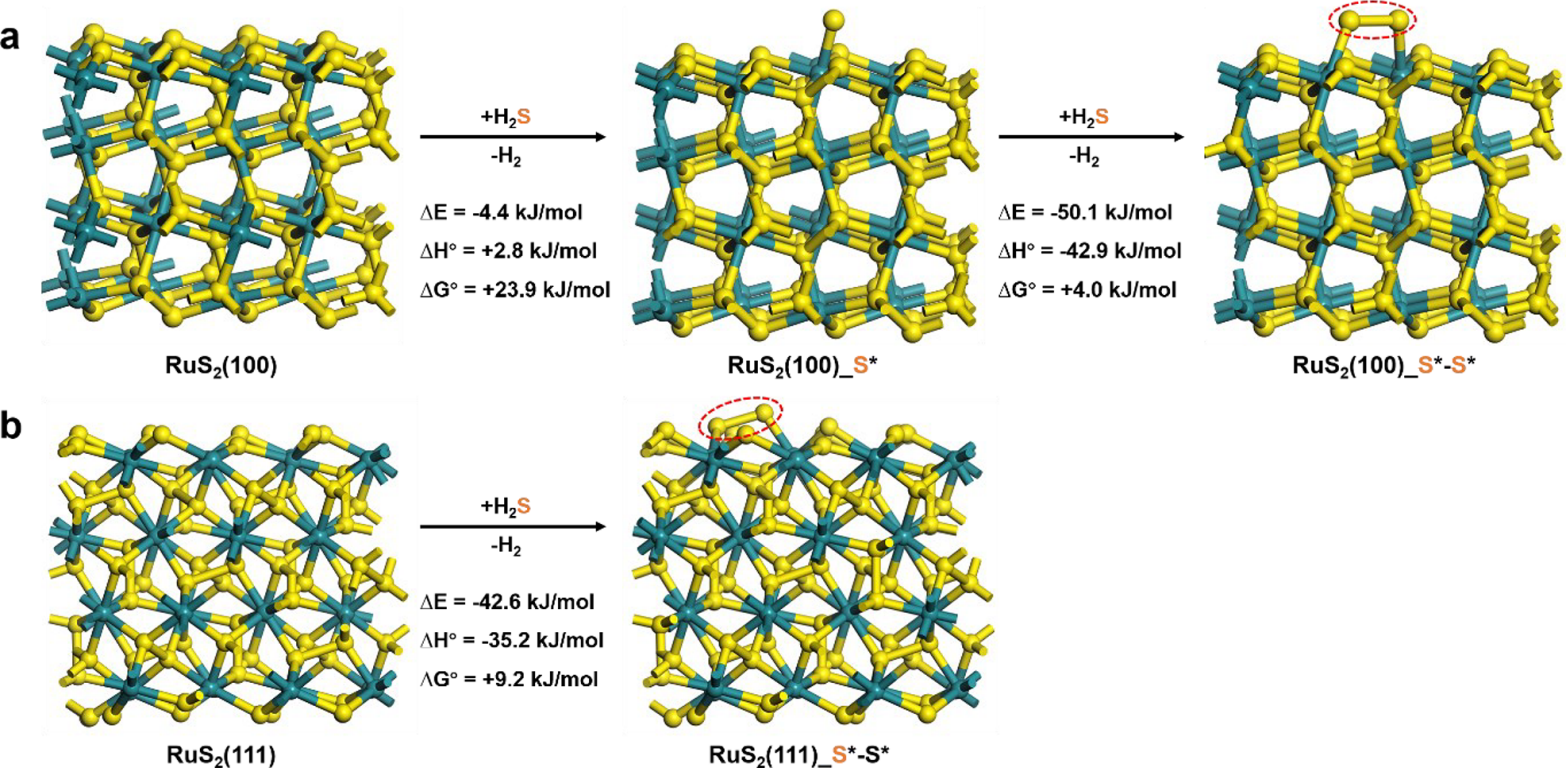
DFT calculations on the energy change of H_2_S decomposition to H_2_ and S* over the (a) RuS_2_(100) and (b) RuS_2_(111) surfaces. The corresponding top views of these structures can be seen in Figure S23.

DFT calculations were utilized to distinguish between the mechanisms (such as competitive vs non-competitive) and to offer a comprehensive representation of the energetic landscape throughout the entire catalytic cycle. Because both mechanisms require H_2_S decomposition to form surface species that assist in C–H bond dissociation, we first assessed the free energy of reaction for this step, which may be formulated as H_2_S + * ⇌ H_2_ + S* or H_2_S + ^1^/_2_*–* ⇌ H_2_ + ^1^/_2_S*–S*. The DFT-computed Δ*G*_rxn,900°C_^°^ is compared for two RuS_2_ model surfaces, RuS_2_(100) and RuS_2_(111), which are the only two low-index facets that the face-centered cubic RuS_2_ nanoparticles can expose (Figure 2d). For the RuS_2_(100) surface, all the surface Ru atoms are in a penta-coordinated state (Figure S23a), and the decomposition of H_2_S on such a Ru site to form H_2_ and an on-top S* monomer (a bridged S cannot be formed) is accompanied by a Δ*G*_rxn,900°C_^°^ of +23.9 kJ/mol (Figure 6a). When the decomposition of the second H_2_S occured on the surface S* monomer, the Gibbs free energy of reaction only slightly increased by 4.0 kJ/mol by forming a S*–S* dimer (Figure 6a), giving an average Δ*G*_rxn,900°C_^°^ of +14.0 kJ/mol (Table 2); this theoretical estimate is more comparable to the experimental value (+10.8 kJ/mol) obtained from regression of measured rate data against the rate equation (eq 1) derived based on the competitive mechanism. For the RuS_2_(111) surface, three-quarters of the surface Ru atoms are in the penta-coordinated state (the others are in the hexa-coordinated state, Figure S23b), and the S atom from the decomposition of H_2_S binds not only to the penta-coordinated Ru site but also to the adjacent S atom, forming a structure analogous to the S*–S* dimer on the RuS_2_(100) surface and giving a similar Δ*G*_rxn,900°C_^°^ of +9.2 kJ/mol (Figure 6b). Notably, an S trimer cannot be formed when binding the third S* in the vicinity of an S*–S* dimer (Figure S24), and the S*–S* dimer should be the most stable structure on both RuS_2_(100) and (111) surfaces, judging from the results of model optimizations (Figure S25).

The close agreement between the theoretical estimates for H_2_S decomposition to gaseous H_2_ and S*–S* dimer (around 0.3 and +10 kJ/mol for *K*_H_2_S,900°C_ and Δ*G*_rxn,900°C_^°^, respectively, Table 2) and the experimental values obtained from regression of measured rate data against the rate equation (eq 1) led us to conclude that the competitive mechanism prevails on RuS_2_ surfaces, and the S*–S* dimers (but not the S* monomers) are the actual working sites for methane activation under HRM conditions. An extended discussion on the active site is presented in the Supporting Information (the passages below Figures S26 and S27). The non-competitive mechanism can be discarded as a major pathway, because the values of *K*_H_2_S,900°C_ (3.2) and Δ*G*_rxn,900°C_^°^ (−11.3 kJ/mol) obtained from regression of measured rate data against the rate equation (eq 2) derived based on such a model are at odds with the theoretical estimates.

We note in passing that, with the accurate knowledge about the thermodynamics of gas phase H_2_S decomposition (Table S10, DFT calculations and HSC Chemistry), it is possible to estimate, in more quantitative terms, the binding thermodynamics of a sulfur dimer (^1^/_2_S_2_(g) + ^1^/_2_*–* ⇌ ^1^/_2_S*–S*) on RuS_2_ surfaces. The experimentally derived Δ*G*_rxn,900°C_^°^ for H_2_S(g) + ^1^/_2_*–* ⇌ H_2_(g) + ^1^/_2_S*–S* (+10.8 kJ/mol) and the Δ*G*_rxn,900°C_^°^ value from HSC Chemistry for H_2_S(g) ⇌ ^1^/_2_S_2_(g) + H_2_(g) (+32.7 kJ/mol) yield a Δ*G*_rxn,900°C_^°^ of −21.9 kJ/mol for ^1^/_2_S_2_(g) + ^1^/_2_*–* ⇌ ^1^/_2_S*–S*; when both values are sourced from DFT calculations (+14.0 or +9.2 kJ/mol for H_2_S + ^1^/_2_*–* ⇌ H_2_ + ^1^/_2_S*–S* on RuS_2_(100) or RuS_2_(111) surfaces, respectively; +28.1 kJ/mol for H_2_S(g) ⇌ ^1^/_2_S_2_(g) + H_2_(g)), Δ*G*_rxn,900°C_^°^ for ^1^/_2_S_2_(g) + ^1^/_2_*–* ⇌ ^1^/_2_S*–S* would be −14.1 or −18.9 kJ/mol, depending on the exposed RuS_2_ surface plane. These Δ*G*_rxn,900°C_^°^ estimates (−14.1 to −21.9 kJ/mol) translate to a moderately strong binding of sulfur dimer at the HRM temperatures (*K*° = 4–10 at 900 °C). As for the enthalpy of such binding, Δ*H*_rxn,900°C_^°^ estimates entirely based on DFT calculations (Tables 2 and S10) indicate an exothermicity of 105 or 120 kJ/mol (per mole of S atom) for RuS_2_(100) or RuS_2_(111) surfaces, respectively.

**Figure 7:**
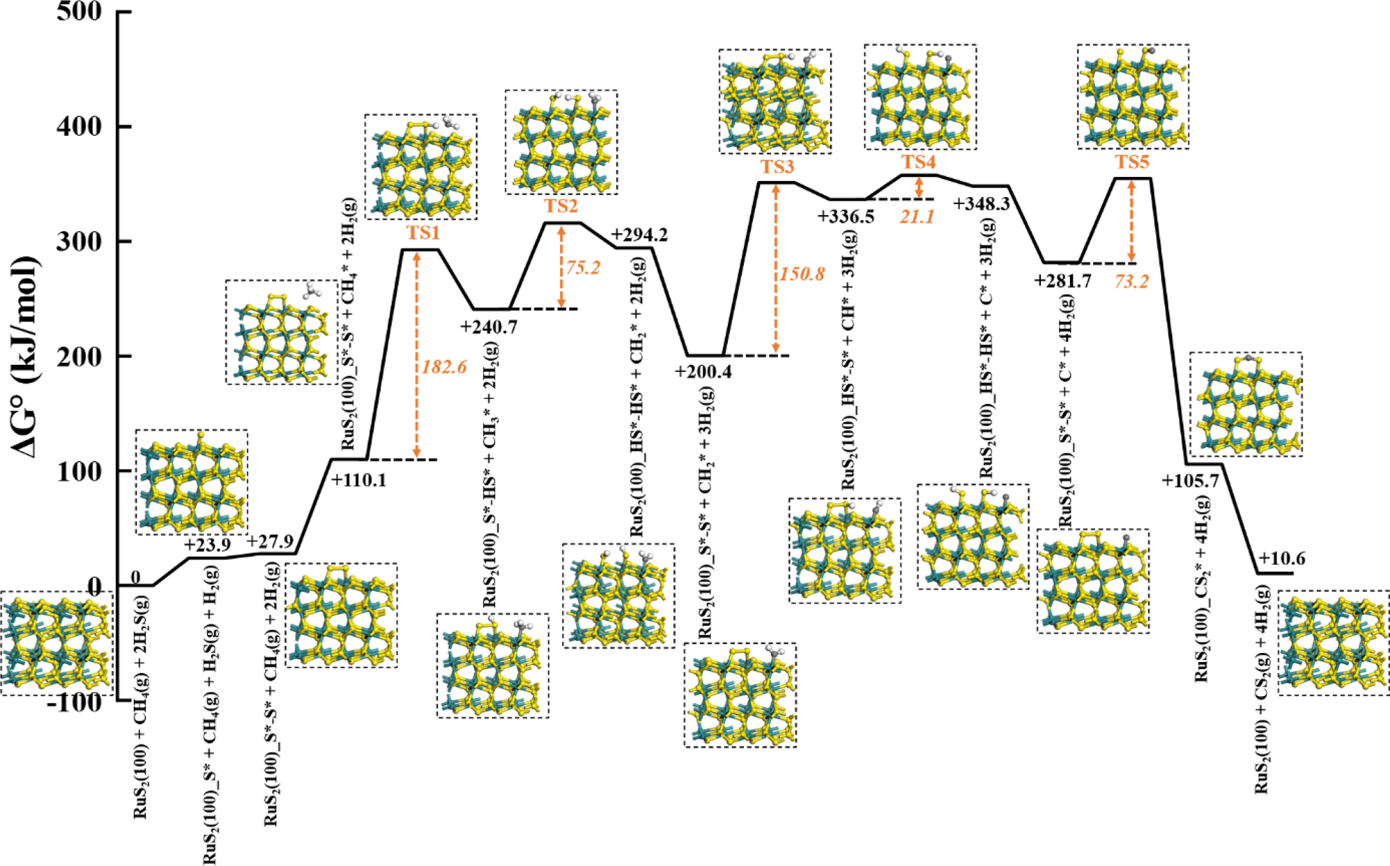
DFT-calculated free energy diagram for the HRM reaction on the RuS_2_(100) surface. The numbers in orange indicate the free energy barriers of C–H scission and CS_2_ formation steps.

The free energy diagram for the complete catalytic cycle was then computed by using the RuS_2_(100) surface (Figure 7). The corresponding enthalpy diagram can be seen in Figure S28. The catalytic cycle starts with the formation of an S*–S* dimer on this surface. Next, CH_4_ is chemisorbed on the surface with a Δ*G*_rxn,900°C_^°^ of +83.2 kJ/mol and a Δ*H*_rxn,900°C_^°^ of −7.8 kJ/mol (Figure S28). The cleavage of the first C–H bond occurs via the assistance of this dimer species, forming CH_3_ on the Ru site and SH; the calculated free energy barrier for this elementary step is 182.6 kJ/mol, which is the highest in the catalytic cycle and thus represents the rate-limiting step, consistent with the experimental finding (Figure 4). The Δ*G*_rxn,900°C_^°^ for this step is also very positive (+130.5 kJ/mol), while the free energy barrier for its reverse is only 52.1 kJ/mol, indicating this step to be reversible, in line with the H/D isotopic exchange data (Figure 3c). The cleavage of the C–H bond in H_3_C* to form H_2_C* (and SH) is characterized by a much smaller Gibbs free energy change (+53.5 kJ/mol) and a much lower barrier (75.2 kJ/mol), indicating that this step is much faster than the first C–H bond scission and, thus, is quasi-equilibrated. The S*–S* dimer structure is recovered via the recombinative desorption of H_2_ from two SH. The third and fourth C–H bond scission steps also exhibit lower free energy barriers and are likely quasi-equilibrated; specifically, the cleavage of the C–H bond of H_2_C* (to form HC* and SH) shows relatively high Δ*G*_rxn,900°C_^°^ (+136.2 kJ/mol) and a free energy barrier (150.8 kJ/mol), while the cleavage of the C–H bond of HC* is associated with a Δ*G*_rxn,900°C_^°^ of +11.7 kJ/mol and a free energy barrier of only 21.1 kJ/mol. The combination of C* and S*–S* dimer to form adsorbed CS_2_, which completes the catalytic cycle, was found to be exergonic (Δ*G*_rxn,900°C_^°^ = −176.1 kJ/mol) with an activation barrier of 73.2 kJ/mol, indicating that it is a quasi-irreversible step, consistent with the results from the CS_2_ co-feeding experiment (Figure 3b). Taken together, the DFT-based mechanistic analysis and the kinetic and isotopic experiments give a coherent picture of HRM catalysis over RuS_2_, which involves the H_2_S-derived S*–S* dimer as the critical enabler of C–H bond scissions. Importantly, the lattice sulfur anions and isolated S* monomers on RuS_2_ surfaces do not seem to play a significant part in the catalytic cycle (Figures S26 and S27).

Although the DFT calculations were performed using a RuS_2_ surface as an example, the similar kinetic behaviors, KIEs, and isotopic exchange patterns observed for other metal or metal sulfides supported on carbon black (as shown in Figures S14, S17, and S29 and Table S7) along with previous reports for bulk oxides, sulfides, and oxysulfides catalysts^49^ collectively indicate the generality of these mechanistic features across a wide spectrum of catalysts. The substantial catalytic activity of CB alone in HRM (but not in other forms of methane reforming) is a surprising finding and implies a similar essential role of dynamically formed sulfur for C–H bond activation on otherwise inert carbon surfaces (Figures S15 and S16); separate detailed DFT assessments are warranted, though the preliminary calculation results (Figure S30) hint that the S*–S* dimer is still the thermodynamically more stable species on the carbon surface.

## Conclusions

Methane reforming with hydrogen disulfide (HRM) represents a crucial process in harnessing the potential of natural gas rich in H_2_S. It offers a pathway to produce CO_*x*_-free H_2_ and valuable sulfur-based chemicals. We show that multiple carbon materials (carbon black, graphene, and carbon nanotubes) and carbon-supported metal catalysts have higher catalytic activities than previously reported catalysts derived from unsupported metal oxides. Carbon-black-supported Ru was identified as one of the most stable and active (on a metal mass basis) catalysts for HRM. Phase, composition, and particle size analyses of carbon-supported catalysts established that the supported metals transformed into their thermodynamically most stable sulfide forms. Carbon supports effectively reduce sintering, which led to more than an order of magnitude higher metal-based rates compared to bulk metal sulfides. The apparent TOF (calculated from the geometric fraction of surface atoms that is in turn estimated using the mean particle size obtained from XRD and TEM measurements) varied by more than 1 order of magnitude across the studied carbon-supported transition metals.

On these carbon-based catalysts, HRM follows a common mechanism, in which H_2_S decomposition and hydrogen combination (or H_2_ dissociation) steps are quasi-equilibrated, whereas successive C–H bond scissions of CH_4_ remain reversible, but not all of them are quasi-equilibrated. DFT calculations showed that the cleavage of the first C–H bond has the highest free energy barrier, and the last C–H bond scission has the lowest barrier. More importantly, theory and experiments collectively establish the dynamically formed and moderately bound dicoordinated sulfur dimers as the direct enabler of methane activation and catalytic turnovers on sulfide and carbon surfaces that are otherwise inherently inert. These extrinsic and reaction-derived active sites are present at a concentration set by the fugacity ratio of H_2_S to H_2_, thus causing the reaction rate to be characteristically inhibited by longer residence times and higher average H_2_ pressures along the catalyst bed, in agreement with our previous study.^49^ This, in turn, suggests that the strength of sulfur binding may serve as a key reactivity descriptor for HRM catalysis. Insufficient sulfur binding can lead to pronounced H_2_ inhibition under typical reaction conditions, while overly strong sulfur binding to the surface may have a detrimental effect on its capacity to facilitate H abstraction from methane, C–S formation, and CS_2_ desorption. The comprehensive analysis of sulfur binding for the different metal sulfides, based on theory and kinetic analysis, as exemplified for Ru/CB catalysts in this work, is important to establish a relationship between sulfur binding and intrinsic reactivity.

## Supporting Information Available

Experimental details (chemicals, catalyst preparation, catalyst characterization, catalytic evaluation, and theoretical calculation), supplementary figures and tables (more thermodynamic, kinetic, and isotopic experimental data, various characterization data of Raman, XRD, XPS, and TEM, and additional theoretical calculation data), and derivation of rate equations (Situation I: competitive mechanism, Section II: non-competitive mechanism, Section III: lattice-S involved mechanism) (PDF)
