# Supplementary material for: Methane–H2S Reforming Catalyzed by Carbon and Metal Sulfide Stabilized Sulfur Dimers
Source: J Am Chem Soc. 2024 Mar 15;146(12):8630–40. doi: 10.1021/jacs.4c00738 (PMC10979457; doi:10.1021/jacs.4c00738)
Supplement: Supplementary file 1 — ja4c00738_si_001.pdf [file ja4c00738_si_001.pdf]

Supporting Information

Methane-H_2S reforming catalyzed by carbon and metal sulfide stabilized sulfur dimers

Yong Wang,[1,2,#,*] Wenru Zhao,[3,#] Xiaofeng Chen,[1] Yinjie Ji,[1,4] Xilei Zhu,[1] Xiaomai Chen,[1] Donghai Mei,[3,*] Hui Shi,[5,*] Johannes A. Lercher[1,4,*]

[1] Department of Chemistry and Catalysis Research Center, Technische Universität München, Lichtenbergstrasse 4, 85748 Garching, Germany

[2] School of Chemical Engineering and Technology, Tianjin University, Tianjin, 300072, P. R. China

[3] School of Materials Science and Engineering, Tiangong University, Tianjin 300387, P. R. China

[4] Institute for Integrated Catalysis, Pacific Northwest National Laboratory, P.O. Box 999, Richland, WA 99352, United States

[5] School of Chemistry and Chemical Engineering, Yangzhou University, Yangzhou, 225002, P. R. China

[#] These authors contributed equally: Yong Wang, Wenru Zhao.

* To whom correspondence should be addressed: yong.wang@tju.edu.cn; dhmei@tiangong.edu.cn; shihui@yzu.edu.cn; johannes.lercher@ch.tum.de

Section S1. Experimental

S1.1 Chemicals, gases, and catalysts

Table S1. Vendor and purity of gases, chemicals, and commercial catalysts used in this work.

Category	Formula	Vendor	Purity
Gas	He	Westfalen AG	99.996%
	N_2	Westfalen AG	99.999%
	H_2	Westfalen AG	99.999%
	CH_4	Westfalen AG	99.995%
	H_2S	Westfalen AG	99.8%
	D_2	Westfalen AG	99%
	CD_4	Eurisotop GmbH	99%
	$CH_4/CS_2/N_2$	Westfalen AG	7.85%/2.08%/90.07%
Liquid	CS_2	Sigma-Aldrich	99%
	$NH_3 \cdot H_2O$	Emplura	32%
Metal Salt	$Ni(NO_3)_2 \cdot 6H_2O$	Aldrich	99.999%
	$(NH_4)_6Mo_7O_{24} \cdot 4H_2O$	Merck	99%
	$RuCl_3 \cdot xH_2O$	Aldrich	38-42% Ru basis
	$RhCl_3 \cdot xH_2O$	Aldrich	38-40% Rh basis
	$PdCl_2$	Aldrich	99%
	H_2WO_4	Aldrich	99%
	NH_4ReO_4	Aldrich	99.999%
	$OsCl_3 \cdot xH_2O$	Aldrich	99%
	$IrCl_3 \cdot xH_2O$	Aldrich	99%
	$H_2PtCl_6 \cdot 6H_2O$	Aldrich	38% Pt basis
Catalyst	RuO_2	Aldrich	99.9%
	TiO_2	Evonik	99.9%
	Carbon Black (CB)	Vulcan	-
	Carbon Nanotube (CNT)	Aldrich	> 98% carbon basis
	Graphene	Aldrich	-

S1.2 Catalyst preparation

Wetness impregnation was used to prepare a TiO_2-supported Ru catalyst and CB-supported noble metal (Ru, Rh, Pd, Re, Os, Ir, and Pt) and non-noble metal (Ni, Mo, and W) catalysts. The corresponding metal precursors are listed in Table S1. Both the weighed metal salt and support (TiO_2 or CB) were dissolved in excess water, and then the mixture was stirred at 25 °C in a fume hood such that almost all water evaporated overnight. Before use, the sample was dried in an oven at 100 °C (static air) for 12 h. The metal loadings for the CB-supported catalysts were around 5 wt% confirmed by ICP-OES.

S1.3 Catalyst characterization

Transmission electron microscopy (TEM) images were acquired on an FEI Tecnai F20 microscope operating at 200 kV. X-ray diffraction (XRD) measurements were conducted on a benchtop X-ray powder diffractometer (Rigaku MiniFlex 600-C) with Cu Kα radiation at 40 kV and 15 mA. MDI Jade 5.0 with database of powder diffraction file (PDF) standards was used to identify the best matched phase structures in each sample. Raman spectra were collected on a Renishaw inVia Reflex Raman spectrometer using a laser source of 532 nm and a power of 0.1 mW to avoid the damage on the sample. N_2 physical adsorption-desorption isotherms were measured at -196 °C on a Surfer gas adsorption porosimeter (Thermo Fisher Scientific). The specific surface area was calculated using the multipoint Brunauer-Emmett-Teller method. X-ray photoelectron spectroscopy (XPS) data were acquired with a Kratos Axis Supra spectrometer at a base pressure below 10^{-8} Torr using monochromatic Al Kα radiation (E = 1486.6 eV), a charge neutralizer, and pass energies of 160 eV (survey spectra) and 80 eV (region spectra). All binding energy values were calibrated using the 1s photoemission peak for carbon at 284.5 eV.

S1.4 Catalytic evaluation

S1.4.1 H_2S reforming of methane

The atmospheric H_2S reforming of methane (HRM) was performed in a quartz tube reactor (65 cm length, 6 mm outer diameter and 4 mm inner diameter) under ambient pressure and temperatures around 900 °C. Flow rates of high-purity gases used for pretreatment and reaction (listed in Table S1) were individually controlled by Bronkhorst mass flow controllers and calibrated by an electronic flowmeter. [**Safety measures and precautions: due to the constant formation of elemental sulfur that deposits as solid particles in cooler sections, a sulfur trap filled with quartz wool was added downstream of the reactor to prevent the six-port valve and the packed column in gas chromatograph (GC) from being blocked. This, however, did not eliminate the entire problem of sulfur deposition and, eventually, complete reactor blockage. In particular, when the long-term stability was evaluated, we were only able to run the reaction for 12 h at most to avoid unattended operation during nighttime, because reactor blockage caused by sulfur deposits would lead to a surge in pressure in the quartz reactor and consequently, undesirable and dangerous incidents such as H_2S leaks.]

Product analysis was performed on a Shimazu 2014 GC equipped with a packed column (HS-Q 80/100, 2.7 m × 2.0 mm ID × 1/8 inch OD, Sulfinert SHDZU 14A NOC) and a thermal

conductivity detector. Gas quantitative calibration was achieved by diluting target gas with N_2 or He. CS_2 quantitative calibration was achieved by bubbling method described in Table S2 and verified with a standard bottle of 7.85% CH_4 and 2.08% CS_2 in N_2. In this work, the forward rate of CH_4 conversion was obtained from the measured net rate by correcting for the approach to equilibrium ($r_f = \frac{r_{net}}{1-\eta}$ and $\eta = \frac{[P_{CS_2}][P_{H_2}]^4}{[P_{CH_4}][P_{H_2S}]^2} \times \frac{1}{K_{eq}}$), and carbon selectivities were calculated based on the amount of CS_2, the only carbon-containing product detected by GC, and the converted amount of CH_4 ($Sel._{C\%} = \frac{n_{CS_2}}{n_{CH_4,conv}}$). Note that this carbon selectivity is conceptually the same as carbon balance, which is around 98% for all catalysts. In a few cases, we verified the hydrogen balance (i.e., the amount of H_2 detected vs. H in converted CH_4 and H_2S) to be nearly 100%, while in most tests the hydrogen balance was not checked because the reliable thermal conductivity detection of H_2 requires N_2 as the carrier gas in which H_2S and CS_2 detection is not as good as in He carrier gas. Sulfur balance was not calculated because the amount of elemental sulfur cannot be quantified.

For the HRM stability tests at 900 °C, 30 mg of quartz wool and a certain amount of catalyst (5 or 20 mg) were packed into a quartz tube reactor. Catalyst was pretreated in a gas of 10% H_2S in H_2 with a total flow of 20 mL/min at 900 °C for 20 min. Then, a feed gas composed of 4 mL/min of CH_4, 12 mL/min of H_2S, and 34 mL/min of He was introduced into the reactor for reaction for 3-8 h.

To obtain the reaction orders in the two reactants of HRM, 30 mg of quartz wool and 20 mg of CB or 5 wt% Ru/CB were packed into a quartz tube reactor and pretreated in a flow of 10% H_2S/H_2 at 900 °C for 20 min. Then, a feed gas of CH_4, H_2S and He was introduced for reaction, and the reaction orders in CH_4 and H_2S were obtained under a total flow of both 50 mL/min. To obtain the reaction order in H_2S, the partial pressure of H_2S was set as 0.08, 0.12, 0.16, 0.24 and 0.32 bar with a constant CH_4 partial pressure of 0.08 bar. To obtain the reaction order in CH_4, the partial pressure of CH_4 was set as 0.06, 0.08, 0.12, 0.16 and 0.24 bar with a constant H_2S partial pressure of 0.24 bar.

For the investigation of product inhibition for HRM, 30 mg of quartz wool and 20 mg of 5 wt% Ru/CB were packed into a quartz tube reactor and pretreated in a gas of 10% H_2S in H_2 at 900 °C for 20 min. Then, a feed gas composed of CH_4, H_2S, and He was introduced to the reactor for reaction at a certain temperature. For H_2 co-feeding at 860, 880, or 900 °C, 2-30 mL/min of H_2 was supplied by replacing the same amount of He to ensure a constant total flow of 50 mL/min and thus a constant partial pressure of CH_4 (0.03-0.12 bar) and H_2S (0.24 bar). For CS_2 co-feeding at 900 °C, a bottle filled with pure CS_2 liquid was installed between the He

mass flow controller and the reactor. The saturated vapor of CS_2 was carried with He flow. By adjusting the temperature of the CS_2 saturator and the He flow rate (Table S2), CS_2 vapor with known partial pressures was fed into the reactor. The He flow was fine-tuned to ensure a constant total flow of 50 mL/min and thus a constant partial pressure of 0.08 bar CH_4 and 0.24 bar H_2S.

Table S2. Partial pressures of CS_2 during CS_2 co-feeding experiments.

Temperature of CS_2 liquid (°C)	Saturated vapor pressure of CS_2 (bar)[a]	CS_2 flow (mL/min)[b]	He flow (mL/min)	Partial pressure of CS_2 (bar)[c]
2	0.185	6.29	27.71	0.126
-8	0.116	3.94	30.06	0.079
-18	0.069	2.35	31.65	0.047
-28	0.039	1.34	32.66	0.027
-38	0.021	0.72	33.28	0.014

[a] Calculated from the Antoine equation: $\log_{10}(P)=A-(B/(T+C))$. For CS_2, A equals to 6.9419; B equals to 1168.62; C equals to 241.54; and P is in mmHg and T is in Celsius ranging from -45 to 69 °C. (Yaws, C. L. and Yang, H. C. To estimate vapor pressure easily. Hydrocarbon Processing, October, 1989, p65.)

[b] Calculated from the equation: $F=P\times34$. P is the saturated vapor pressure of CS_2 in column 2. 34 with a unit of mL/min is the total flow of CS_2 and He.

[c] Calculated from the equation: $P'=F/50$. F is the flow of CS_2 in column 3. 50 with a unit of mL/min is the total flow of CS_2, He, CH_4, and H_2S.

For the investigation of kinetic isotope effect (CH_4/CD_4) for HRM, 30 mg of quartz wool and 20 mg of CB or 5 wt% Ru/CB diluted with 100 mg of quartz sand were packed into a quartz tube reactor and pretreated in a flow of 10% H_2S/H_2 at 900 °C for 20 min. Then, a feed gas composed of CH_4 (0.04 or 0.08 bar), H_2S (0.08 or 0.24 bar), H_2 (0-0.6 bar), and He was introduced for reaction at 900 °C for around 20 min. After that, CH_4 was replaced by CD_4 with the same flow rate for reaction for the same time. It should be noted that the conversion factor of CD_4 is different from that of CH_4 on the same mass flow controller. Finally, CH_4 with the same flow rate was switched back to check the recovery of activity.

S1.4.2. Isotope scrambling experiments

For the H/D isotopic exchange experiments during HRM, 30 mg of quartz wool and 20 mg of catalyst dilution with 100 mg of quartz sand were packed into a quartz tube reactor and pretreated in a gas of 10% H_2S in H_2 at 900 °C for 20 min. Then, a feed gas composed of CH_4, H_2S, D_2, and N_2 was introduced for reaction. Four samples (5 wt% Ru/CB, Pt/CB, Ir/CB, and pure CB) were tested at 900 °C under different flow conditions as summarized in Table S3, and

Ru/CB was also tested at 880 and 860 °C under those flow conditions. A portable mass spectrometer (Pfeiffer Vacuum OmniStar GSD 320) was used for the quantitative analysis of the isotopomer distributions of hydrogen (H_2, HD, D_2), hydrogen disulfide (H_2S, HDS, D_2S), and methane (CH_4, CH_3D, CH_2D_2, CHD_3, CD_4). Because there are overlaps between some molecular ion peaks and some fragment peaks from higher mass molecules (i.e., H_2S and DS from both D_2S and HDS), subtraction should be done to get an accurate isotopomer distribution, especially for the more complicated methane molecules. For this purpose, fragmentation patterns of each molecule need to be obtained in advance. Unfortunately, most D-labelled gases cannot be available, including HD, HDS, D_2S, CH_3D, CH_2D_2, and CHD_3. Thus, we first tested the fragmentation patterns of H_2, D_2, H_2S, CH_4, and CD_4 on our mass spectrometer, and then made a reasonable estimation for the fragmentation patterns of the other molecules by comparing our data with those from NIST database and two references (Dibeler, V. H. and Mohler, F. L. Mass spectra of the deuteromethanes. Journal of Research of National Bureau of Standards, 1950, 45, 441-444; Dibeler, V. H. and Rosenstock, H. M. Mass spectra and metastable transitions of H_2S, HDS, and D_2S. The Journal of Chemical Physics, 1963, 39, 3106-3111.)

Table S3. Flow conditions used for H/D isotopic exchange experiments during HRM.

Serie	Flow rate (mL/min)					D/H[a]	Partial pressure (bar)		
	CH_4	H_2S	D_2	N_2	Total		CH_4	H_2S	D_2
1	2	6	2	15	25	0.2	0.08	0.24	0.08
2	4	12	4	30	50	0.2	0.08	0.24	0.08
3	6	18	6	45	75	0.2	0.08	0.24	0.08
4	2	6	4	13	25	0.4	0.08	0.24	0.16
5	4	12	8	26	50	0.4	0.08	0.24	0.16
6	6	18	12	39	75	0.4	0.08	0.24	0.16
7	4	12	12	22	50	0.6	0.08	0.24	0.24
8	2	12	4	32	50	0.25	0.04	0.24	0.08
9	4	4	4	38	50	0.33	0.08	0.08	0.08

[a]The molar ratio of D to H that used for the calculation of binomial distribution.

S1.5 Theoretical calculation

All periodic density functional theory (DFT) calculations were performed with mixed Gaussian and plane wave basis sets implemented in the CP2K code.[1] The core electron was represented by norm-conserving Goedecker-Teter-Hutter pseudopotentials,[2-4] and the valence

electron wavefunctions were expanded in a triple-zeta basis set with polarization functions[5] along with an auxiliary plane wave basis set with an energy cutoff of 400 Ry. The generalized gradient approximation exchange-correlation functional of Perdew, Burke and Enzerhof (PBE) was used.[6] Test calculations showed that the total energy change of the system was negligible (<0.01 eV) when the maximum force convergence criteria of 0.001 Hartree/Bohr was used. Each reaction intermediate structure was optimized with the Broyden-Fletcher-Goldfarb-Shanno (BGFS) algorithm with the SCF convergence criteria of 1.0×10^{-8} a.u. To account for the long-range van der Waals interactions, the DFT-D3 scheme with an empirical damped potential term was added to the electronic energy.[7] The climbing image elastic band (CI-NEB) method[8-9] was used to determine the transition states of all elementary reaction steps in the bond scissions of C-H and the bond recombination of C-S. The transition state was located with five intermediate images along the reaction pathway between the initial and final states. Each identified transition state was further confirmed by the vibrational frequency analysis, in which only one imaginary frequency was found at the transition state.[10]

The Gibbs free energy including zero-point energy (ZPE), internal energy, and entropy along the reaction pathway was calculated using the standard statistical mechanics method,[11-13] that is

$$G = H - TS = E_{elec} + E_{ZPE} + U - TS$$

where E_{elec} is the electronic term, E_{ZPE} is the ZPE contribution, U is the internal energy, S is the entropy, and T (=1173 K, typical experimental condition) is the temperature. The electronic term (E_{elec}) is directly calculated from DFT calculations. The ZPE contribution is given by

$$\mathrm{ZPE} = \sum_i \frac{hv_i}{2}$$

where h and v_i are Planck's constant and vibrational frequencies. The vibrational frequencies are calculated in the framework of the localized harmonic oscillator approximation with a displacement of 0.01 Å. In the vibrational frequency calculation, only mobile reactants/intermediates were considered while other atoms on the RuS_2 (100) and (111) frameworks were fixed. We used the VASPKIT code for post-processing of the CP2K calculated data.[14]

For the RuS_2 (100) and (111) surfaces, a p(4×4) supercell slab with four atomic layers was used in the optimization. Upon optimization, the bottom two layers of the surface slab are fixed while the atoms in the top two layers and the adsorbate are allowed to move during the calculations of elementary steps. A vacuum of 10 Å in the z direction of the simulation box was inserted for each surface model.

References

1. VandeVondele, J.; Krack, M.; Mohamed, F.; Parrinello, M.; Chassaing, T.; Hutter, J., QUICKSTEP: Fast and Accurate Density Functional Calculations Using a Mixed Gaussian and Plane Waves Approach. *Comput. Phys. Commun.* **2005,** *167*, 103-128.
2. Goedecker, S.; Teter, M.; Hutter, J., Separable Dual-space Gaussian Pseudopotentials. *Phys. Rev. B: Condens. Matter* **1996,** *54* (3), 1703-1710.
3. Krack, M.; Parrinello, M., All-electron Ab-initio Molecular Dynamics. *Phys. Chem. Chem. Phys.* **2000,** *2* (10), 2105-2112.
4. Hartwigsen, C.; Goedecker, S.; Hutter, J., Relativistic Separable Dual-space Gaussian Pseudopotentials from H to Rn. *Phys. Rev. B* **1998,** *58* (7), 3641-3662.
5. VandeVondele, J.; Hutter, J., Gaussian Basis Sets for Accurate Calculations on Molecular Systems in Gas and Condensed Phases. *J. Chem. Phys.* **2007,** *127* (11), 114105.
6. Perdew, J. P.; Burke, K.; Ernzerhof, M., Generalized Gradient Approximation Made Simple. *Phys. Rev. Lett.* **1996,** *77* (18), 3865-3868.
7. Grimme, S.; Antony, J.; Ehrlich, S.; Krieg, H., A Consistent and Accurate Ab Initio Parametrization of Density Functional Dispersion Correction (DFT-D) for the 94 Elements H-Pu. *J. Chem. Phys.* **2010,** *132* (15), 154104.
8. Henkelman, G.; Uberuaga, B. P.; Jónsson, H., A Climbing Image Nudged Elastic Band Method for Finding Saddle Points and Minimum Energy Paths. *J. Chem. Phys.* **2000,** *113* (22), 9901-9904.
9. Mills, G. J., H.; Schenter, G. K., Reversible Work Transition State Theory: Application to Dissociative Adsorption of Hydrogen. *Surf. Sci.* **1994,** *324*, 305-337.
10. Grybos, R.; Benco, L.; Bucko, T.; Hafner, J., Molecular Adsorption and Metal-support Interaction for Transition-metal Clusters in Zeolites: NO Adsorption on Pd(n) (n=1-6) Clusters in Mordenite. *J. Chem. Phys.* **2009,** *130* (10), 104503.
11. John, M.; Alexopoulos, K.; Reyniers, M.-F.; Marin, G. B., Reaction Path Analysis for 1-butanol Dehydration in H-ZSM-5 Zeolite: Ab Initio and Microkinetic Modeling. *J. Catal.* **2015,** *330*, 28-45.
12. Gao, F.; Mei, D.; Wang, Y.; Szanyi, J.; Peden, C. H., Selective Catalytic Reduction over Cu/SSZ-13: Linking Homo- and Heterogeneous Catalysis. *J. Am. Chem. Soc.* **2017,** *139* (13), 4935-4942.
13. Mei, D.; Lercher, J. A., Mechanistic Insights into Aqueous Phase Propanol Dehydration in H-ZSM-5 Zeolite. *AIChE J.* **2017,** *63* (1), 172-184.
14. Wang, V.; Xu, N.; Liu, J.-C.; Tang, G.; Geng, W.-T., VASPKIT: A User-friendly Interface Facilitating High-throughput Computing and Analysis Using VASP Code. *Comput. Phys. Commun.* **2021,** *267*, 108033.

Section S2. Supplementary Figures and Tables

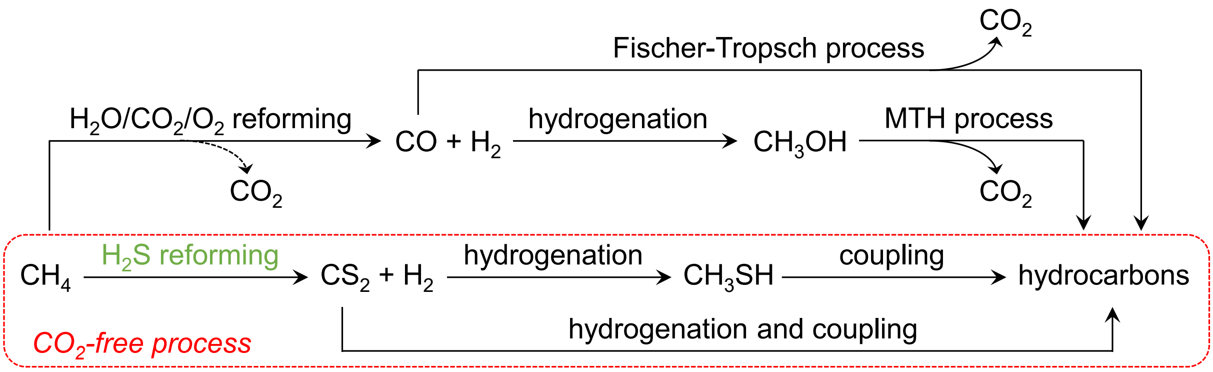

Figure S1. A process for methane utilization that nominally avoids CO_2 emission, starting with H_2S reforming of methane (HRM).

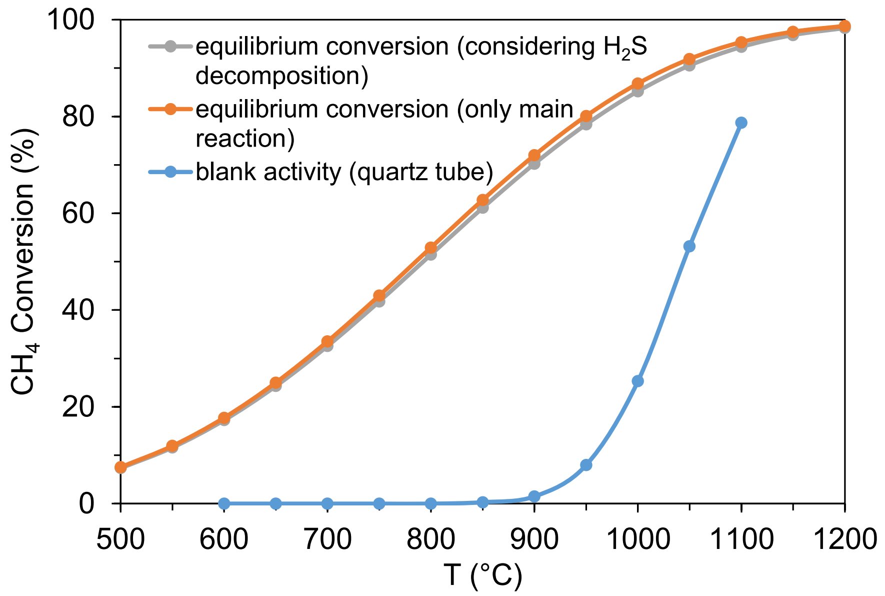

Figure S2. Equilibrium conversions of CH_4 and blank activity of quartz tube reactor under a feed gas of 4 mL/min of CH_4, 12 mL/min of H_2S, and 34 mL/min of He at ambient pressure.

a

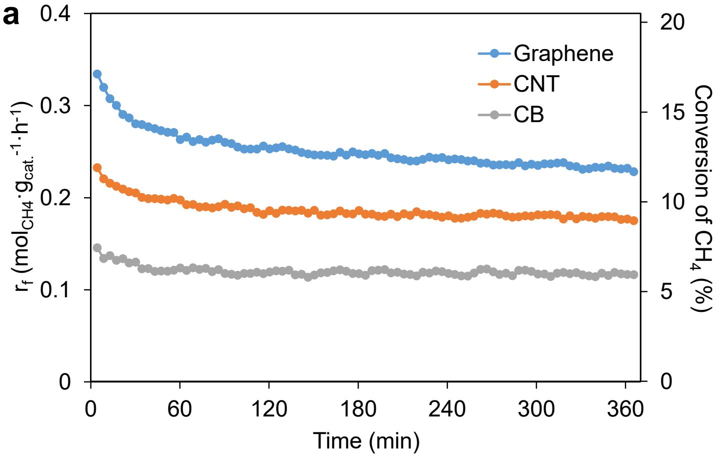

b

Sample	Surface area (m^2/g)	Pore volume (cm^3/g)
Graphene	445	2.87
CNT	288	0.57
CB	218	0.28

Figure S3. (a) Catalytic stability of HRM over three carbon materials at 900 °C for 6 h. Pretreatment conditions: 5 mg of catalyst, 20 mL/min of 10% H_2S in H_2 (1 bar), 900 °C, 20 min. Reaction conditions: 0.08 bar CH_4 and 0.24 bar H_2S in He (1 bar), 48 $L_{CH4}\cdot g_{cat}^{-1}\cdot h^{-1}$. (b) Surface area and pore volume of graphene, CNT, and CB.

It can be seen that graphene deactivated more markedly than the other two carbon allotropes. In addition to showing the greatest stability, CB is also cost-effective and more easily granulated, which can avoid the pressure drop when charging more catalysts.

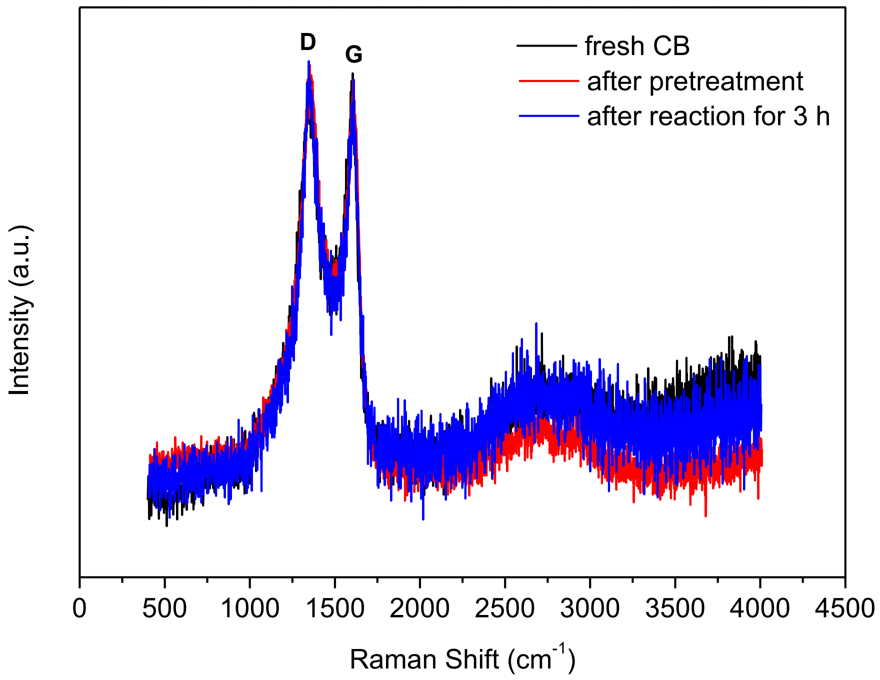

Figure S4. Raman spectra of the fresh CB, the sample after pretreatment, and the sample after reaction for 3 h. Pretreatment and reaction conditions can be seen in the caption of Figure 1.

As shown above, there is almost no difference in these Raman spectra, indicating that CB is robust enough under our harsh reaction conditions. Moreover, elemental analysis shows that the S contents (wt%) were determined to be quite low, around 0.55 %, 0.85 %, and 1.0% for the fresh CB, the sample after pretreatment, and the sample after reaction for 3 h, respectively (Table S4). The relatively obvious increase of the S content occurred in the pretreatment stage (50 min in total), probably due to the reaction between H_2S and certain kinds of the surface oxygenated groups or defects, or the deposition of sulfur upon H_2S decomposition. These data illustrate the resistance of Vulcan XC72 to H_2S atmosphere at high temperatures.

Table S4. Elemental analysis on the content of S in the fresh CB, the sample after pretreatment, and the sample after reaction for 3 h.

Sample	S content (1st test)	S content (2nd test)
fresh CB	0.52 wt%	0.57 wt%
after pretreatment	0.90 wt%	0.78 wt%
after reaction for 3 h	0.98 wt%	1.01 wt%

Table S5. Comparison of TOF at 900 °C across different CB-supported catalysts (metal loadings all at 5 wt%).[a]

Catalyst	Phase	Mean Size (nm)	Dispersion (%)	Rate ($mol_{CH4}\cdot g_{cat.}^{-1}\cdot h^{-1}$)[b]	TOF (s^{-1})
Pt/CB	PtS	41±3	2.3-2.6	0.3957 (0.2782)	12.4±0.7
Pd/CB	PdS	45±10	1.8-2.8	0.4035 (0.2860)	7.6±1.6
Rh/CB	Rh_2S_3	23±4	3.7-5.3	0.4321 (0.3146)	4.1±0.7
Ru/CB	RuS_2	18±2	5.0-6.3	0.4852 (0.3677)	3.7±0.4
Ir/CB	Ir	10±1	9.1-11.1	0.4303 (0.3128)	3.3±0.3
Os/CB	Os	24±4	3.6-5.0	0.2433 (0.1258)	3.2±0.5
Ti/CB	$Ti_{2.45}S_4$	40±4	2.3-2.8	0.2677 (0.1502)	2.7±0.3
Ni/CB	NiS and Ni_3S_4	40±10	2.0-3.3	0.2940 (0.1765)	2.3±0.6
Re/CB	ReS_2	13±2	6.7-9.1	0.2574 (0.1399)	1.9±0.3
Mo/CB	MoS_2	8×19	5.3-12.5	0.4015 (0.2840)	2.0±0.8
W/CB	WS_2	8×19	5.3-12.5	0.2267 (0.1092)	1.5±0.6

[a] Pretreatment conditions: 5 mg of catalyst, 20 mL/min of 10% H_2S in H_2 (1 bar), 900 °C, 20 min. Reaction conditions: 0.08 bar CH_4 and 0.24 bar H_2S in He (1 bar), 48 $L_{CH4}\cdot g_{cat.}^{-1}\cdot h^{-1}$. The average particle sizes in these used catalysts were calculated from the XRD patterns (Figure S9) using the Scherrer equation, and the corresponding dispersion (D) was estimated from the diameter (d) using D = 1/d. Then, the turnover frequency (TOF) of each catalyst was estimated based on the end-point activity of the stability tests shown in Figure 1 in the main text.

[b] The data in the parentheses correspond to the rate after subtracting the catalytic activity of CB (0.1175 $mol_{CH4}\cdot g_{cat.}^{-1}\cdot h^{-1}$), considering that the surface area of 5 wt% metal with such large particle sizes (10-50 nm) is negligible compared to that of CB (1-5 m^2/g_{CB} vs. 218 m^2/g).

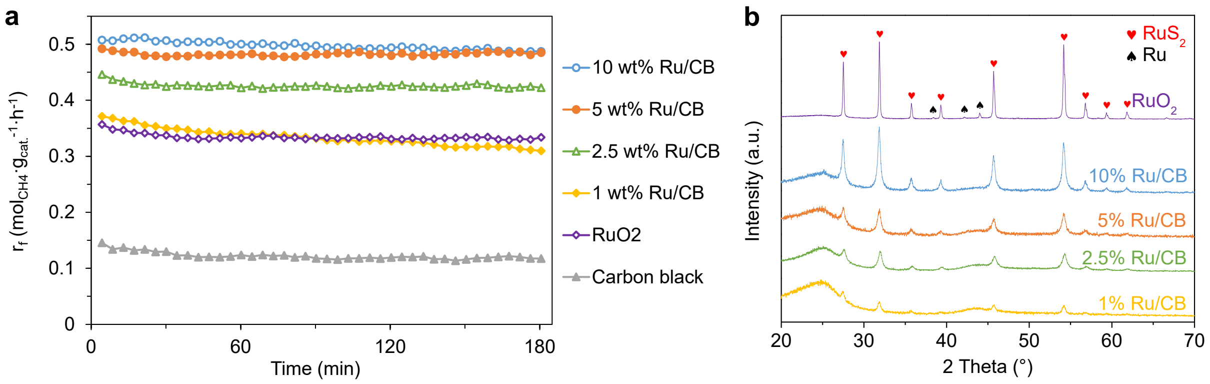

Figure S5. (a) Catalytic stability over RuO_2 and Ru/CB with different loadings at 900 °C for 3 h. (b) XRD patterns of these samples after reaction. Pretreatment conditions: 5 mg of catalyst, 20 mL/min of 10% H_2S in H_2 (1 bar), 900 °C, 20 min. Reaction conditions: 0.08 bar CH_4 and 0.24 bar H_2S in He (1 bar), 48 $L_{CH4}\cdot g_{cat.}^{-1}\cdot h^{-1}$.

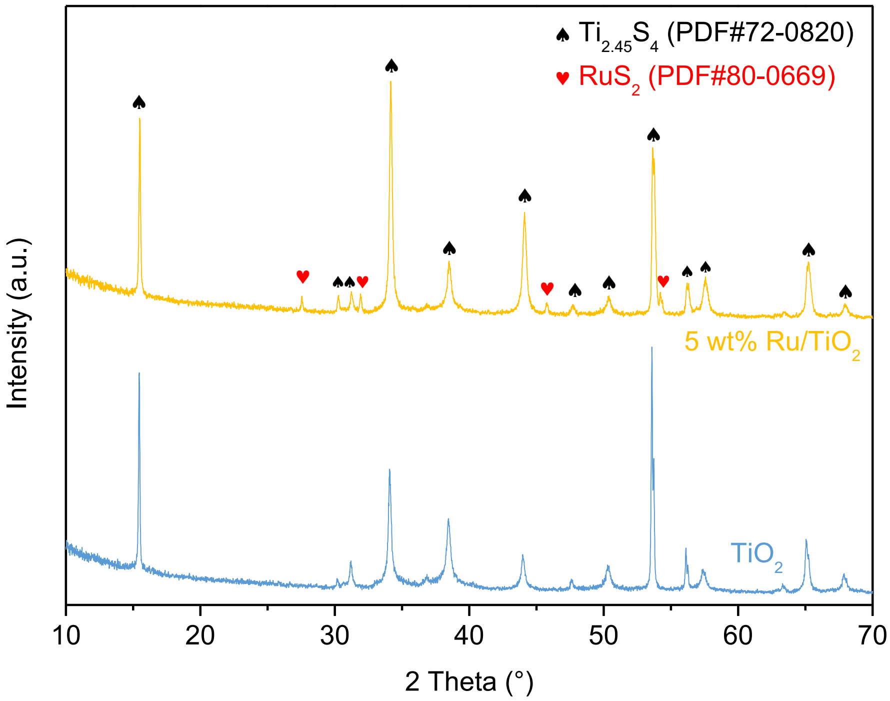

Figure S6. XRD patterns of TiO_2 and 5 wt% Ru/TiO_2 after reaction at 900 °C for 8 h (corresponding to the tested materials in Figure 2a).

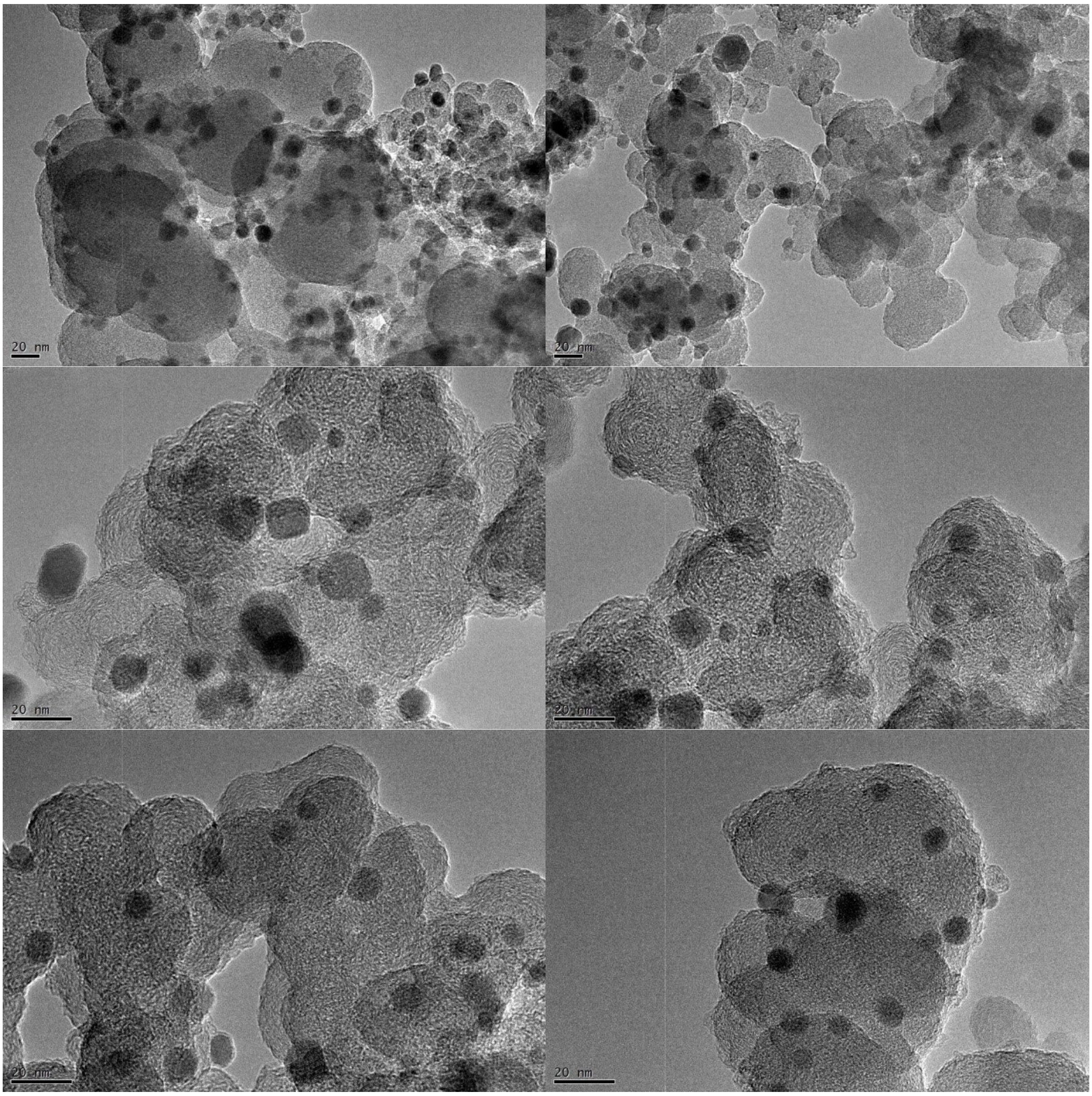

Figure S7. TEM images of the spent 5 wt% Ru/CB (corresponding to the tested material in Figure 2a).

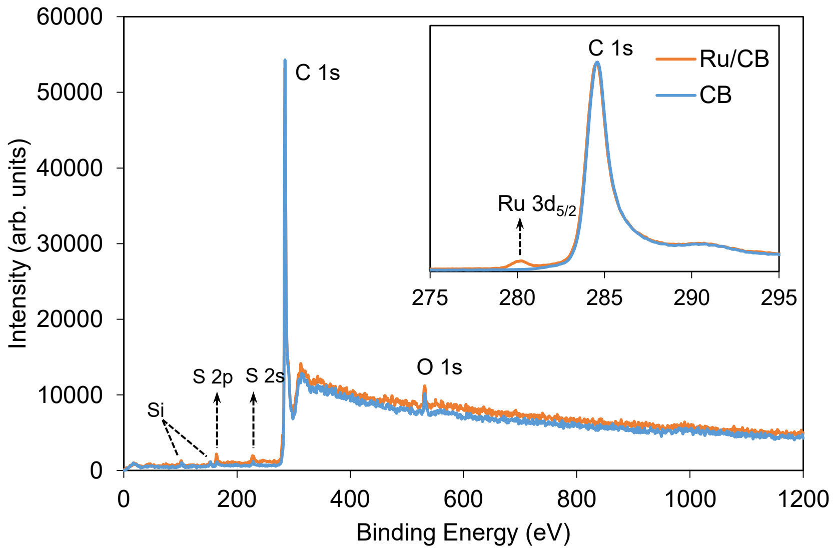

Figure S8. XPS spectra of the spent 5 wt% Ru/CB and CB (corresponding to the tested materials in Figure 2a). The Si signals (Si 2p and 2s from left to right) come from the quartz sand residues.

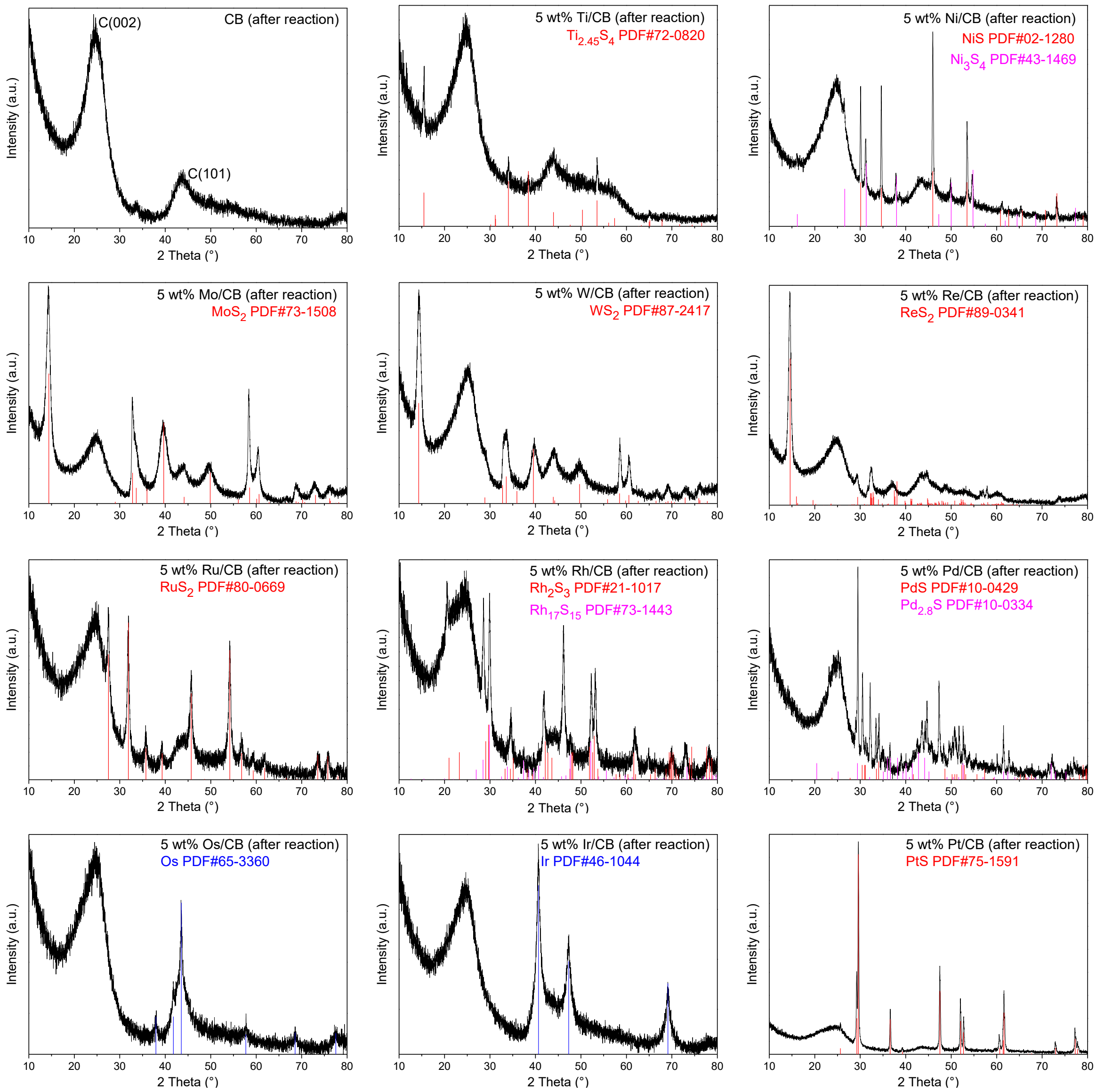

Figure S9. XRD patterns of CB-supported metal catalysts after reaction at 900 °C for 3 h (corresponding to the tested catalysts in Figure 1).

Table S6. Phase analysis of spent catalysts by XRD and equilibrium constants (K_{eq}) of sulfidation reaction of noble metals with H_2S at 900 °C calculated by HSC Chemistry.

Catalyst	Phase[a]	Mean Size (nm)[b]	Sulfidation Reaction	$K_{eq,900°C}$
Ru/CB	RuS_2	18±2	$Ru + 2H_2S(g) \rightarrow RuS_2 + 2H_2(g)$	65.67
Rh/CB	Rh_2S_3	23±4	$Rh + 1.5H_2S(g) \rightarrow 0.5Rh_2S_3 + 1.5H_2(g)$	7.893
Re/CB	ReS_2	13±2	$Re + 2H_2S(g) \rightarrow ReS_2 + 2H_2(g)$	6.812
Pt/CB	PtS	41±3	$Pt + H_2S(g) \rightarrow PtS + H_2(g)$	0.8697
Pd/CB	PdS	45±10	$Pd + H_2S(g) \rightarrow PdS + H_2(g)$	0.5485
Ir/CB	Ir	10±1	$Ir + 2H_2S(g) \rightarrow IrS_2 + 2H_2(g)$	0.0953
Os/CB	Os	24±4	$Os + 2H_2S(g) \rightarrow OsS_2 + 2H_2(g)$	0.0857

[a] XRD patterns can be seen in Figure S9. For Rh/CB and Pd/CB, only the main phases were listed. For Ir/CB and Os/CB, their metal phases in bulk do not mean there is no sulfide adlayer, especially under reaction conditions.

[b] The mean particle size was estimated from the diffraction peaks of multiple crystal planes based on the Scherrer equation.

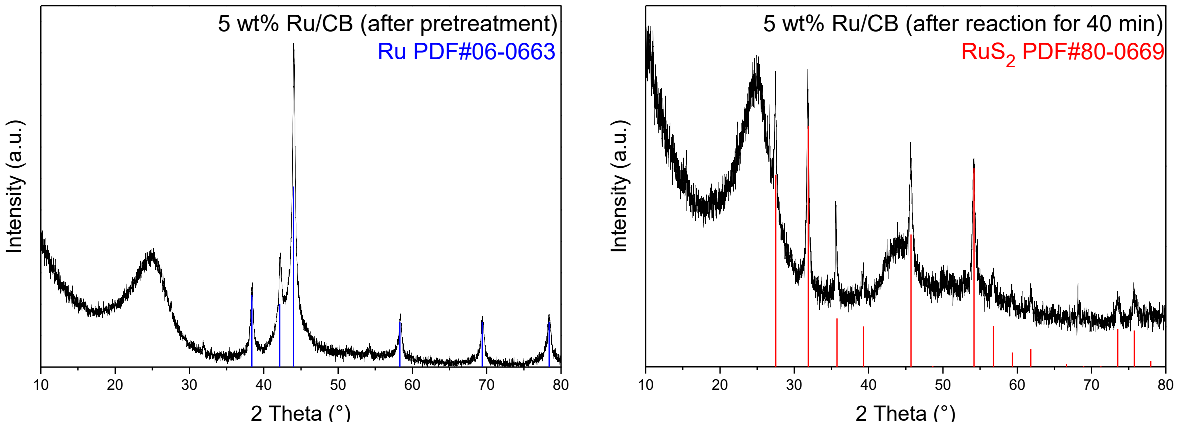

Figure S10. XRD pattern of Ru/CB after pretreatment in 10% H_2S/H_2 at 900 °C for 20 min (50 min in total including the heating process) and Ru/CB after reaction at 900 °C for 40 min.

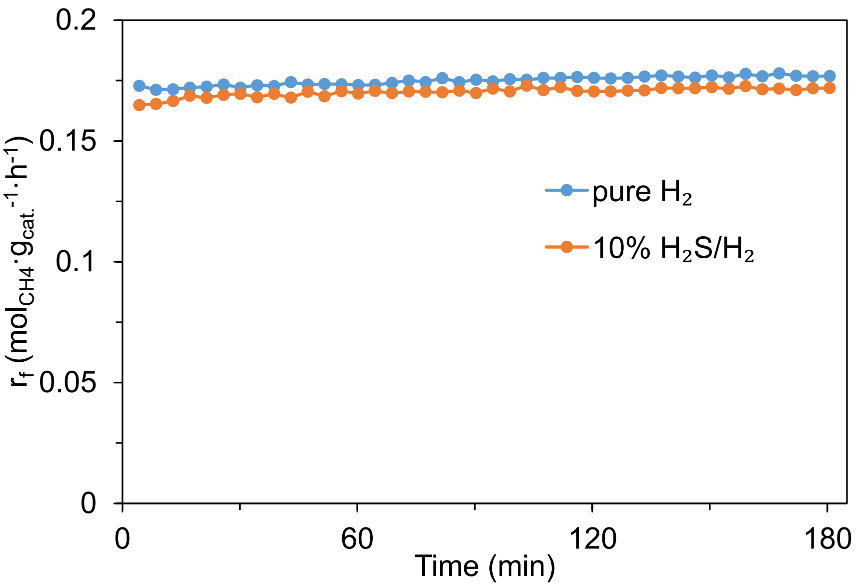

Figure S11. Influence of pretreatment atmosphere on the catalytic performance of HRM over 5 wt% Ru/CB. Pretreatment conditions: 20 mg of catalyst diluted with 100 mg of quartz sand, 20 mL/min of pure H_2 or 10% H_2S in H_2 (1 bar), 900 °C, 20 min. Reaction conditions: 0.08 bar CH_4 and 0.24 bar H_2S in He (1 bar), 12 $L_{CH4}\cdot g_{cat.}^{-1}\cdot h^{-1}$, 900 °C. The orange data points are extracted from Figure 2a in the main text.

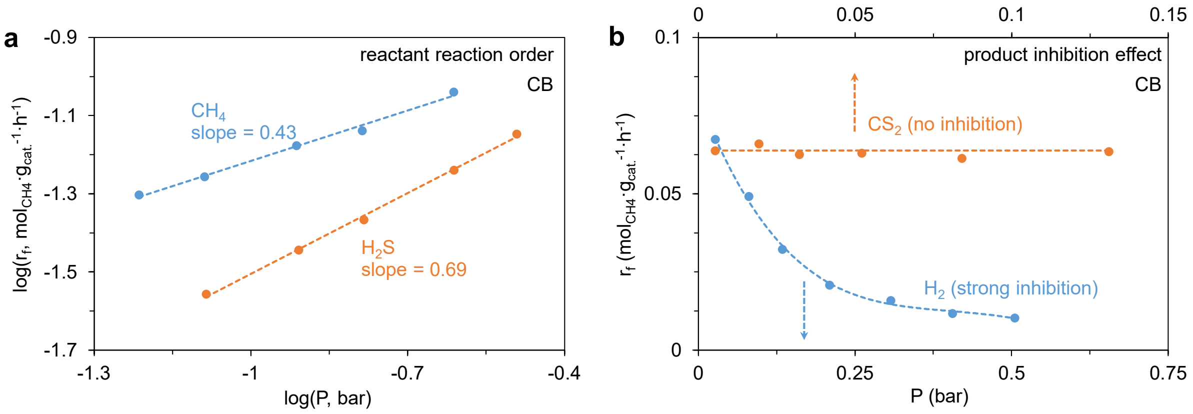

Figure S12. (a) Reaction orders of CH_4 and H_2S over CB at a certain pressure of 0.24 bar H_2S and 0.08 bar CH_4, respectively. 150 $L \cdot g_{cat.}^{-1} \cdot h^{-1}$, 900 °C. (b) Influence of co-feedings of H_2 and CS_2 on the forward rate of CH_4 conversion over CB. 0.08 bar CH_4 and 0.24 bar H_2S in He (1 bar), 12 $L_{CH4} \cdot g_{cat.}^{-1} \cdot h^{-1}$, 900 °C. Pretreatment conditions: 20 mg of CB, 20 mL/min of 10% H_2S in H_2 (1 bar), 900 °C, 20 min.

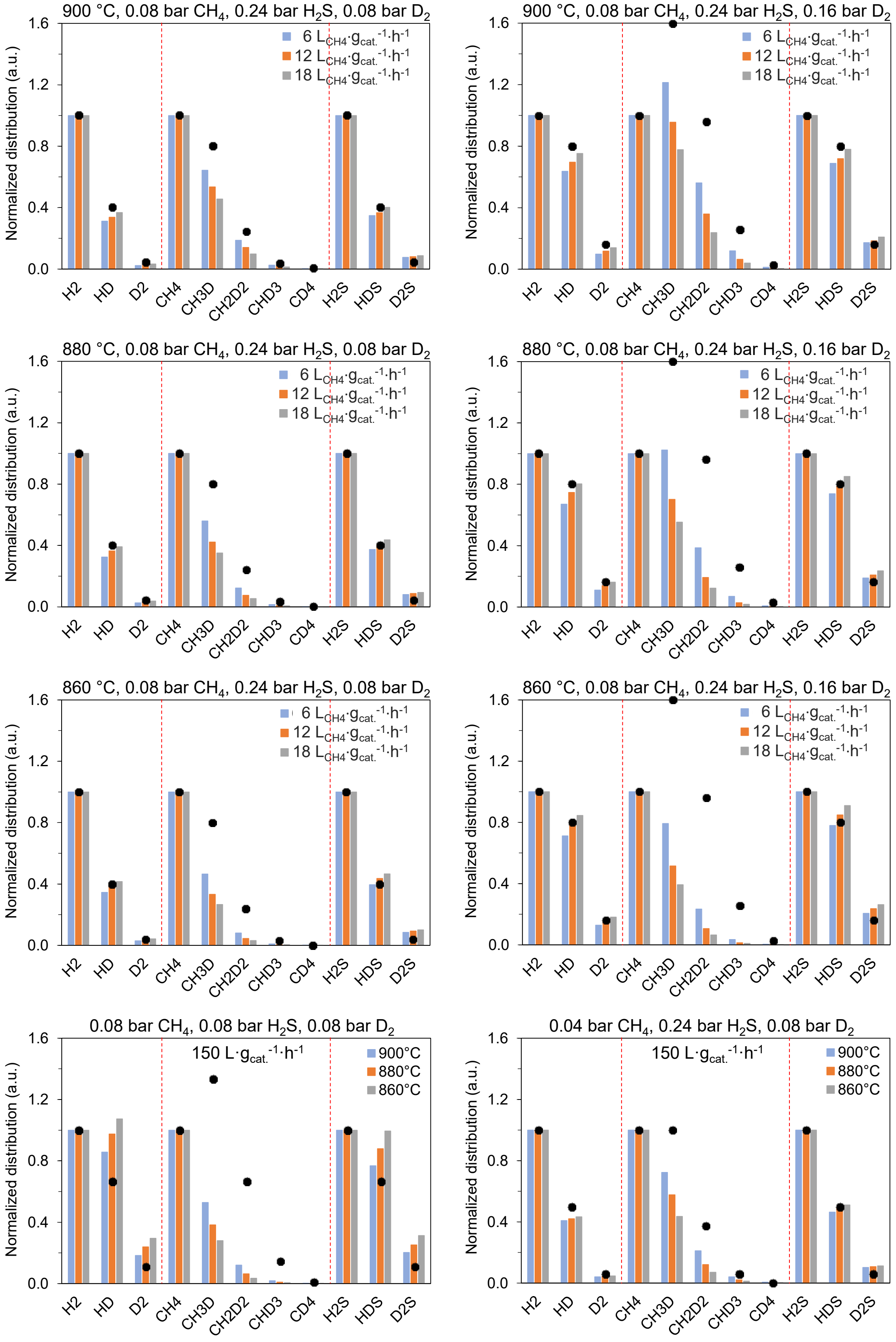

Figure S13. Normalized isotopomer distributions of hydrogen (H_2, HD, D_2), methane (CH_4, CH_3D, CH_2D_2, CHD_3, CD_4) and hydrogen disulfide (H_2S, HDS, D_2S) during reactions of CH_4, H_2S, and D_2 mixtures over 5 wt% Ru/CB under various conditions.

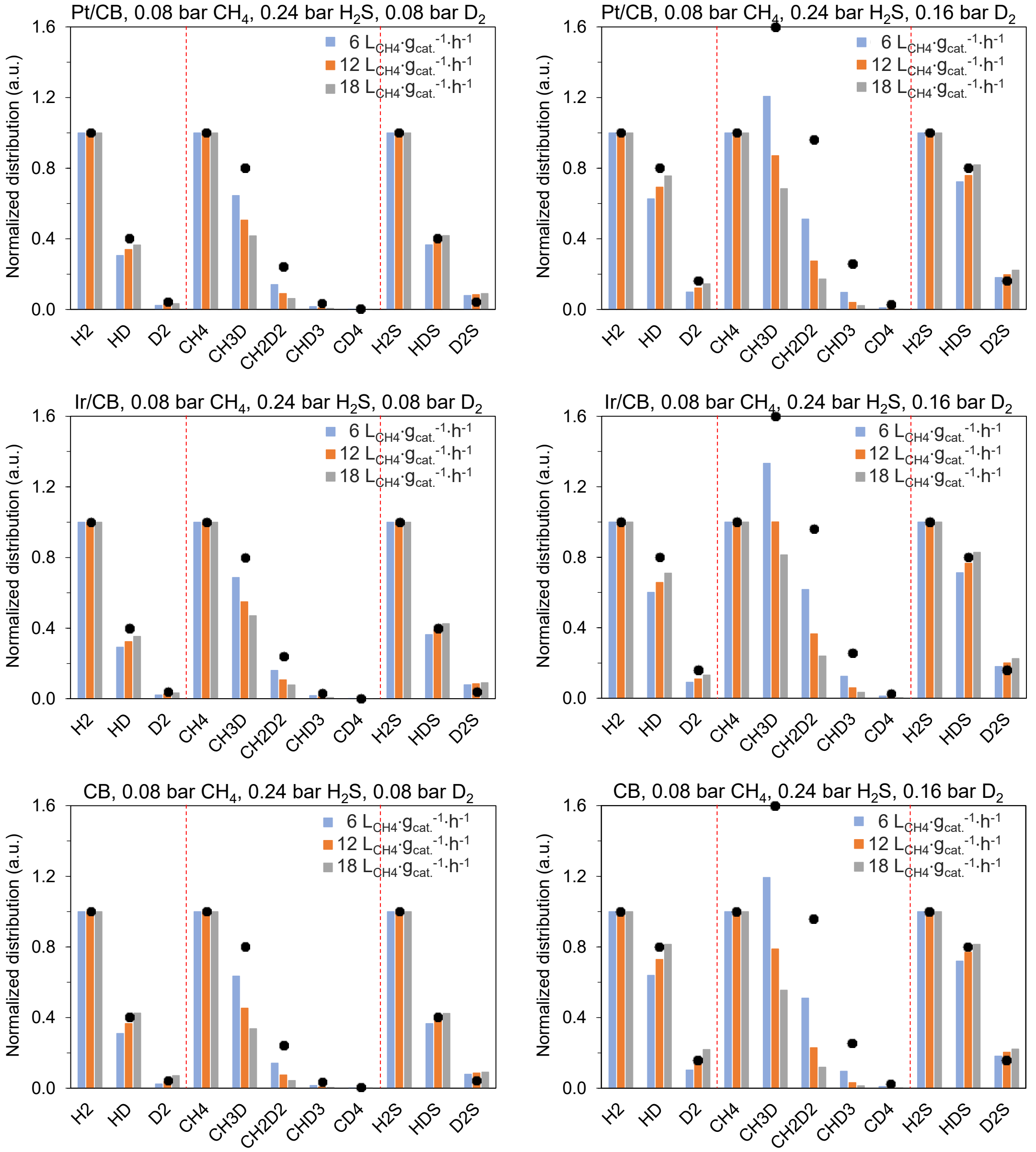

Figure S14. Normalized isotopomer distributions of hydrogen (H_2, HD, D_2), methane (CH_4, CH_3D, CH_2D_2, CHD_3, CD_4) and hydrogen disulfide (H_2S, HDS, D_2S) during reactions of CH_4, H_2S, and D_2 mixtures over 5 wt% Pt/CB (upper panel), 5 wt% Ir/CB (middle panel), and CB (lower panel) at 900 °C.

Table S7. Kinetic isotope effects for HRM over 5 wt% Ru/CB and CB at 900 °C.

Catalyst	P (bar)			GHSV	Conversion (%)		r_{CH4}/r_{CD4}
	CH_4 or CD_4	H_2S	H_2	($L·g_{cat.}^{-1}·h^{-1}$)	CH_4	CD_4	
Ru/CB	0.04	0.24	0	150	44.2	37.2	1.19
	0.08	0.08	0	150	24.8	19.3	1.28
	0.08	0.24	0	75	48.2	41.8	1.15
	0.08	0.24	0	150	33.3	28.2	1.18
	0.08	0.24	0	225	30.3	23.6	1.28
	0.08	0.24	0.12	150	22.9	16.6	1.38
	0.08	0.24	0.2	150	14.9	10.0	1.49
	0.08	0.24	0.5	150	9.9	6.3	1.55
	0.08	0.24	0.6	150	6.7	4.1	1.63
CB	0.08	0.24	0	150	12.5	10.2	1.23
	0.08	0.24	0.12	150	6.3	4.6	1.37
	0.08	0.24	0.2	150	4.1	2.7	1.52

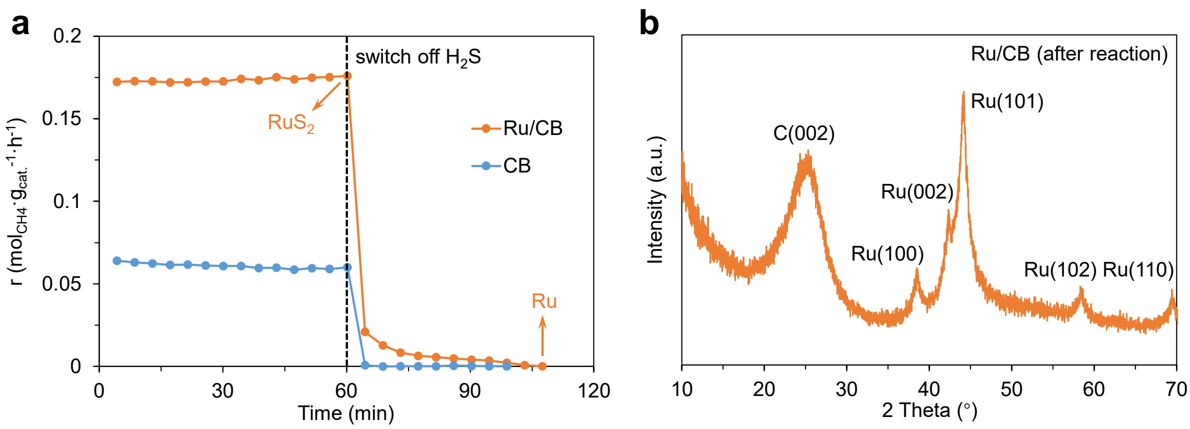

Figure S15. (a) H_2S transient measurement during the steady state operation of HRM over 5 wt% Ru/CB and CB. Pretreatment conditions: 20 mg of catalyst, 20 mL/min of 10% H_2S in H_2 (1 bar), 900 °C, 20 min. Reaction conditions: 0.08 bar CH_4 and 0.24 bar H_2S in He (1 bar), 12 $L_{CH4}\cdot g_{cat.}^{-1}\cdot h^{-1}$, 900 °C. (b) XRD pattern of the Ru/CB catalyst after reaction in (a).

For Ru/CB, when switching off H_2S during HRM, the methane conversion rate plunged by 88% and then gradually reduced to zero after 45 min (Figure S15a). After reaction, the RuS_2 phase before switching (Figure S10) was reduced back to metallic Ru (Figure S15b), indicating that the converted methane after switching arose from the reaction between CH_4 and RuS_2 and probable carbon deposition on the surface of formed Ru NPs. By contrast, for CB, there was no residual activity of methane conversion after switching off H_2S (the blank activity of the quartz tube reactor shown in Figure S2 had been subtracted), indicating that methane cannot be activated on the surface of CB in the absence of H_2S.

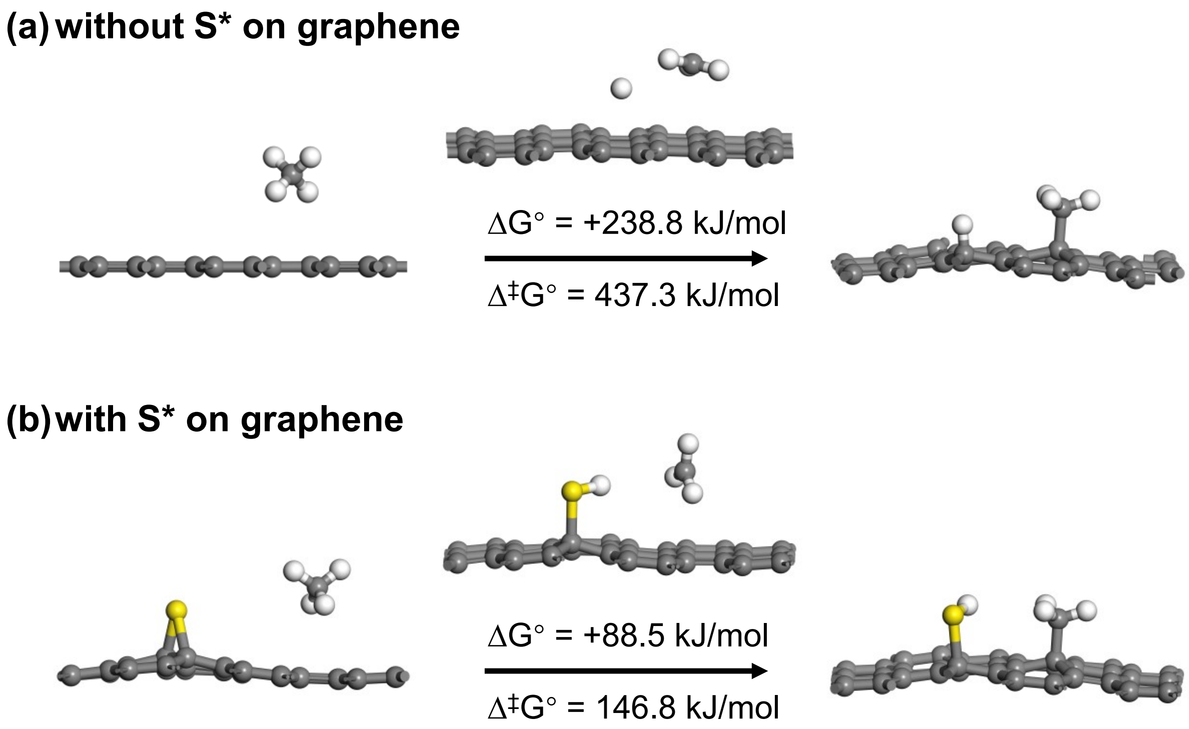

Figure S16. The DFT-calculated Gibbs free energy change and energy barrier for the cleavage of the first C-H bond of CH_4 on (a) a clean graphene surface and (b) a S*-anchored graphene surface at 900 °C.

The cleavage of the first C-H bond of CH_4 is hard to proceed on a clean graphene surface, as the Gibbs free energy change is as high as 238.8 kJ/mol at 900 °C, and the energy barrier is also very high (437.3 kJ/mol). In comparison, on a S*-anchored graphene surface, the Gibbs free energy change of this process reduces to 88.5 kJ/mol at 900 °C, and the energy barrier is much low (146.8 kJ/mol), showing the key role of S* in the activation of methane.

It should be noted that, the model of single-layer graphene cannot perfectly represent the real surface structure of CB, and only one S adatom is not sufficient to complete the catalytic cycle. The true active site of CB should be very complex and diverse, considering different kinds of defects and edges of carbon matrix and unknow configurations of S atoms, which is hardly to be exhausted by theoretical modeling. Thus, in this work, we mainly focused on the modeling of RuS_2 which is simpler and clearer as shown in the main text.

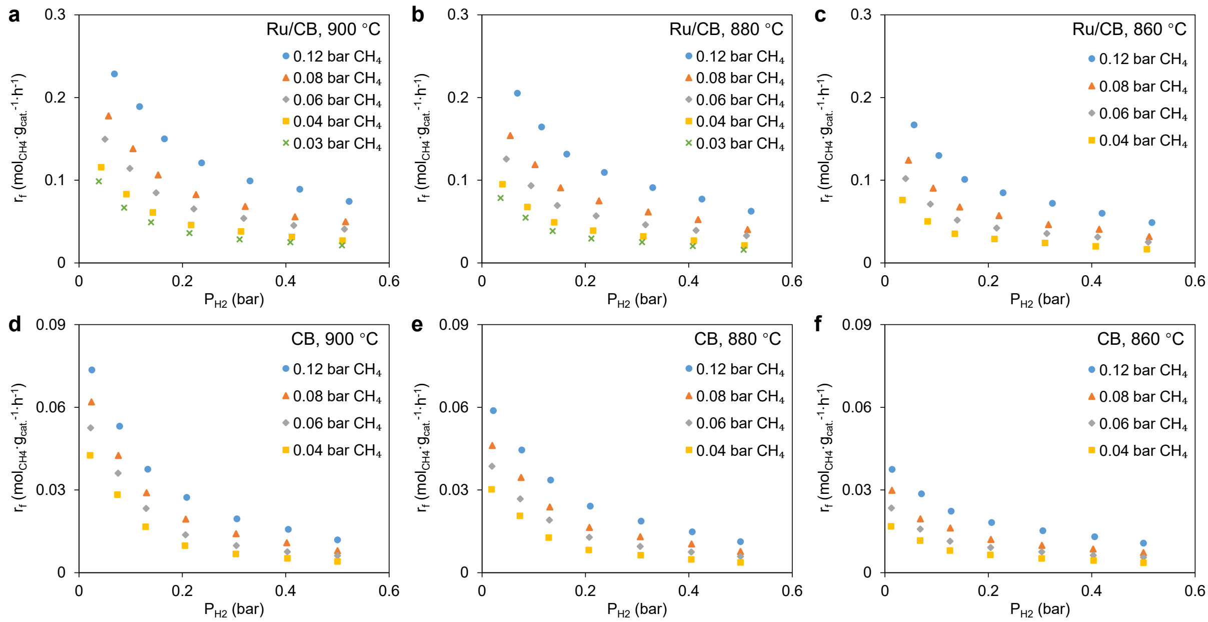

Figure S17. Influence of co-feeding H_2 on the forward rate of CH_4 conversion in HRM over (a-c) 5 wt% Ru/CB and (d-e) CB at different temperatures. Pretreatment conditions: 20 mg of catalyst diluted with 100 mg of quartz sand, 20 mL/min of 10% H_2S in H_2 (1 bar), 900 °C, 20 min. Reaction conditions: 0-0.5 bar H_2, 0.03-0.12 bar CH_4 and 0.24 bar H_2S in He (1 bar), 150 $L{\cdot}g_{cat.}^{-1}{\cdot}h^{-1}$. The partial pressure of H_2 plotted here is the average pressure of the inlet (co-fed) and outlet (generated + co-fed) pressures of H_2.

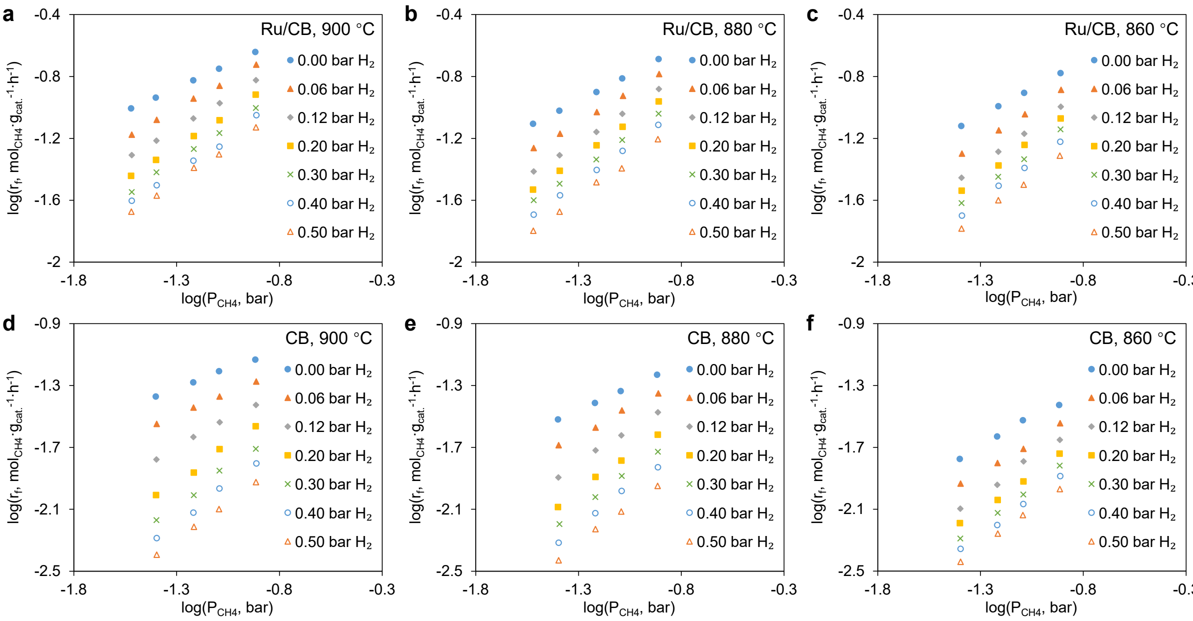

Figure S18. Plots for obtaining reaction orders in CH_4 over (a-c) 5 wt% Ru/CB and (d-f) CB obtained from the H_2 co-feeding experiments shown in Figure S17.

Table S8. Reaction orders in CH_4 over 5 wt% Ru/CB and CB under different partial pressures of co-fed H_2 (0-0.5 bar) obtained from Figure S18.

H_2 (bar)	0	0.06	0.12	0.20	0.30	0.40	0.50
Ru/CB, 900 °C	0.61	0.74	0.80	0.86	0.88	0.90	0.90
Ru/CB, 880 °C	0.68	0.79	0.87	0.93	0.92	0.95	0.97
Ru/CB, 860 °C	0.71	0.85	0.95	0.97	0.98	0.99	0.97
CB, 900 °C	0.50	0.57	0.73	0.93	0.97	1.01	0.97
CB, 880 °C	0.60	0.70	0.86	0.96	0.98	1.02	0.99
CB, 860 °C	0.73	0.80	0.94	0.93	0.97	0.98	0.98

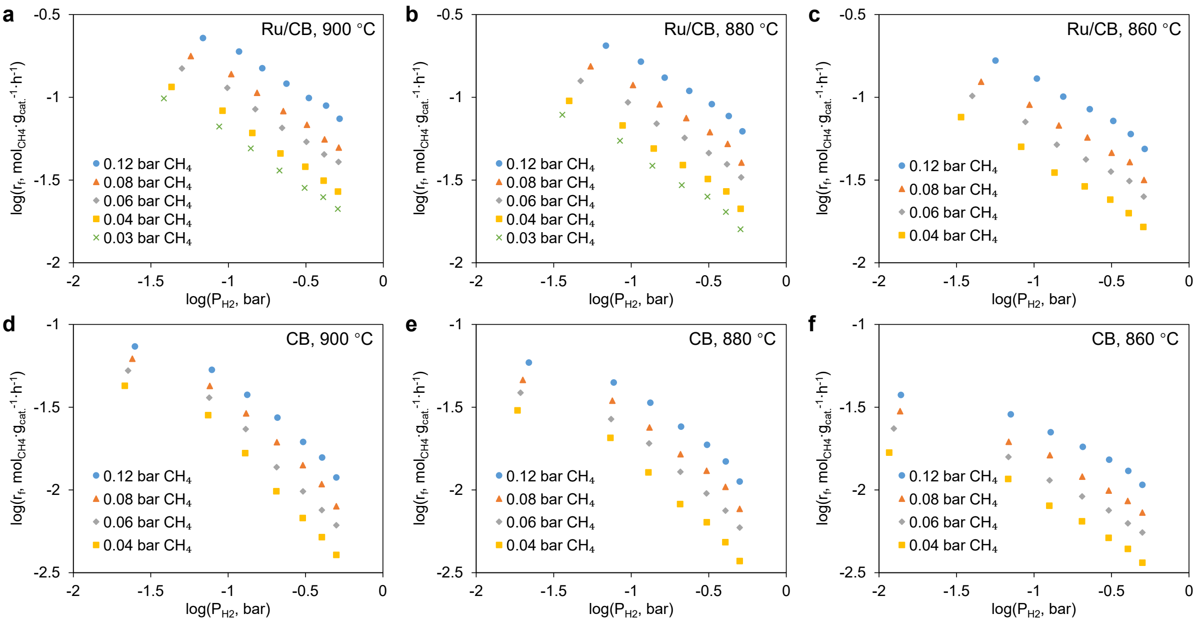

Figure S19. Plots for obtaining reaction orders in H_2 over (a-c) 5 wt% Ru/CB and (d-e) CB obtained from the H_2 co-feeding experiments shown in Figure S17.

Table S9. Reaction orders in H_2 over 5 wt% Ru/CB and CB under different partial pressures of CH_4 (0.03-0.12 bar) and H_2 (0.06-0.5 bar) obtained from Figure S19.

H_2 (bar)	0.06-0.2					0.2-0.5				
CH_4 (bar)	0.03	0.04	0.06	0.08	0.12	0.03	0.04	0.06	0.08	0.12
Ru/CB, 900 °C	-0.68	-0.69	-0.69	-0.66	-0.63	-0.60	-0.62	-0.57	-0.63	-0.59
Ru/CB, 880 °C	-0.67	-0.62	-0.59	-0.58	-0.57	-0.69	-0.68	-0.64	-0.73	-0.70
Ru/CB, 860 °C		-0.54	-0.53	-0.58	-0.59		-0.64	-0.58	-0.67	-0.67
CB, 900 °C		-1.04	-0.96	-0.78	-0.68		-0.99	-0.91	-0.99	-0.92
CB, 880 °C		-0.89	-0.71	-0.73	-0.61		-0.89	-0.87	-0.84	-0.85
CB, 860 °C[a]		-0.54	-0.50	-0.44	-0.42		-0.62	-0.56	-0.55	-0.58

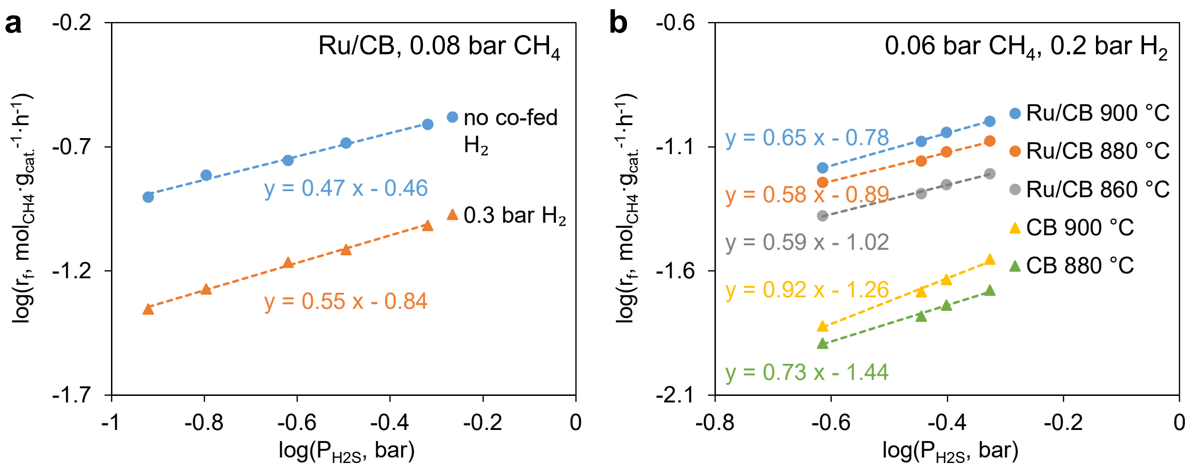

Figure S20. Reaction orders in H_2S over 5 wt% Ru/CB and CB under various conditions. Pretreatment conditions: 20 mg of catalyst diluted with 100 mg of quartz sand, 20 mL/min of 10% H_2S in H_2 (1 bar), 900 °C, 20 min. Reaction conditions: 0-0.3 bar H_2, 0.08 or 0.06 bar CH_4 and 0.08-0.48 bar H_2S in He (1 bar), 150 $L\cdot g_{cat.}^{-1}\cdot h^{-1}$.

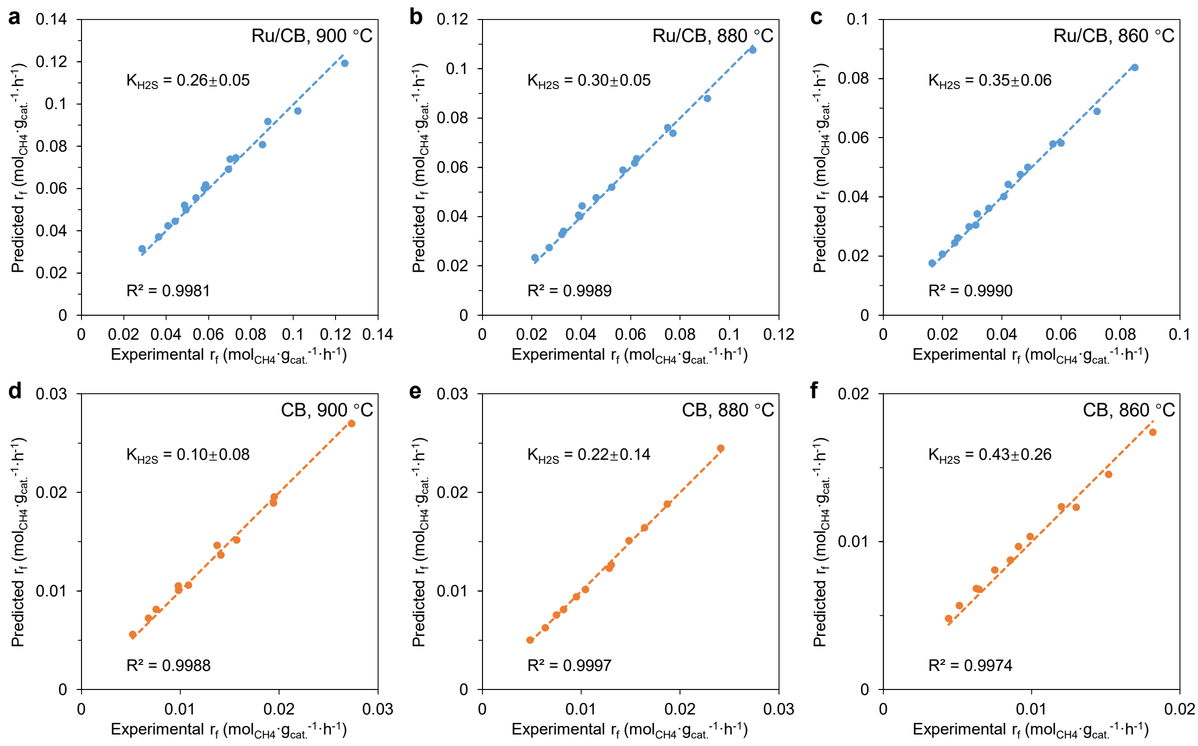

Figure S21. Parity plots of the predicted and measured forward CH_4 conversion rates over (a-c) 5 wt% Ru/CB and (d-e) CB at different temperatures. The rates were predicted by fitting to the rate equation of $r_{f1} = k \times [CH_4] \times [H_2]^n \times \frac{K_{H_2S}\frac{[H_2S]}{[H_2]}}{\left(1+K_{H_2S}\frac{[H_2S]}{[H_2]}\right)^2}$, giving the regressed value of n between -0.1 and 0 for each and that of K_{H_2S} shown in each panel with uncertainties representing 95% confidence interval. The data in the H_2 pressure range of 0.2-0.6 bar shown in Figure S17 were used for fitting.

The differences in K_{H_2S} obtained at different temperatures are quite small compared to the error margin. The same situation can be seen in Figure S22. Thus, it is challenging to get a precise value of enthalpy change for the equilibria of $H_2S + * \rightleftharpoons H_2 + S*$ by applying the van't Hoff equation. However, by and large, the regressed K_{H_2S} value slightly increases with decreasing the temperature, indicating a mildly exothermic reaction for this process.

table

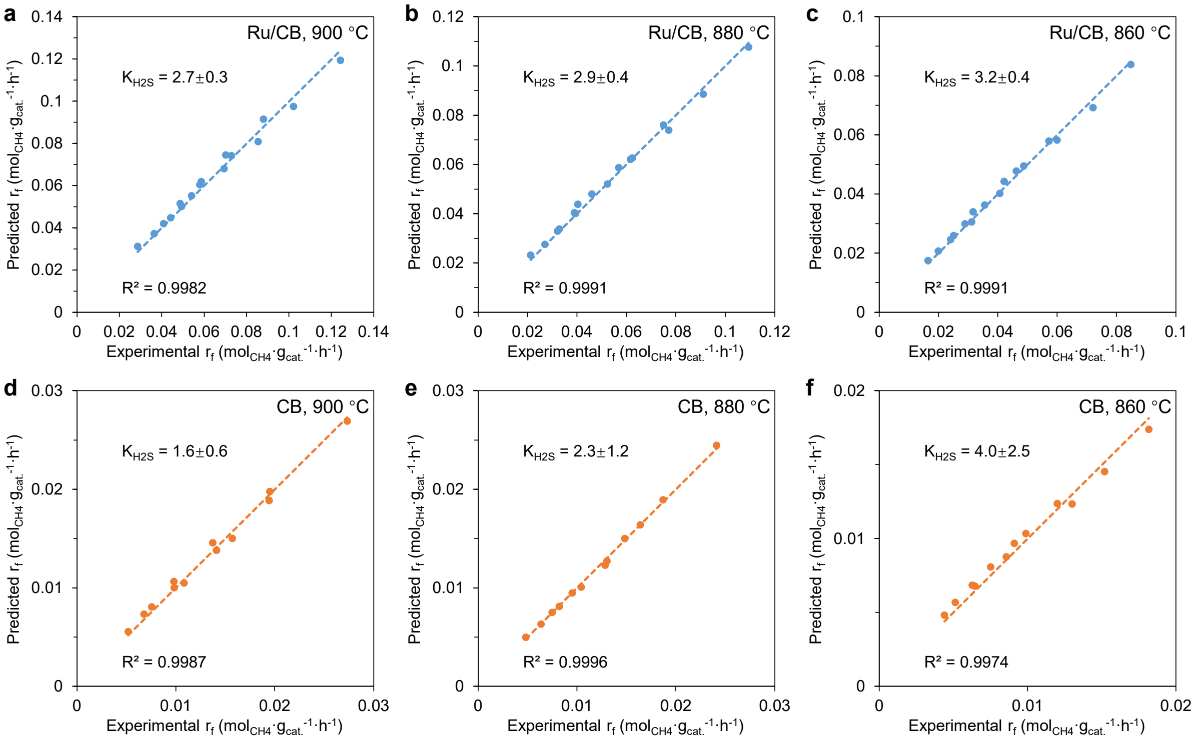

Figure S22. Parity plots of the predicted and measured forward CH_4 conversion rates over (a-c) 5 wt% Ru/CB and (d-e) CB at different temperatures. The rates were predicted by fitting to the rate equation of $r_{f2} = k \times [CH_4] \times [H_2]^n \times \frac{\left(K_{H_2S}\frac{[H_2S]}{[H_2]}\right)^2}{\left(1+K_{H_2S}\frac{[H_2S]}{[H_2]}\right)^2}$, giving the regressed value of n between -0.1 and 0 for each and that of K_{H_2S} shown in each panel with uncertainties representing 95% confidence interval. The data in the H_2 pressure range of 0.2-0.6 bar shown in Figure S17 were used for fitting.

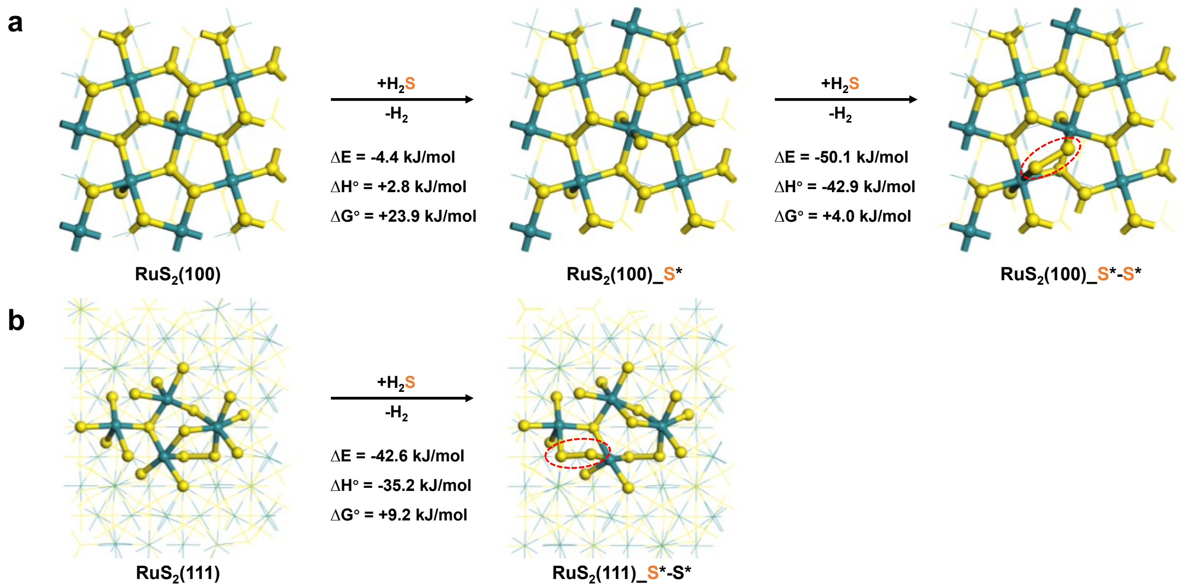

Figure S23. Top views of the structures based on (a) $RuS_2(100)$ and (b) $RuS_2(111)$ surfaces shown in Figure 6 in the main text.

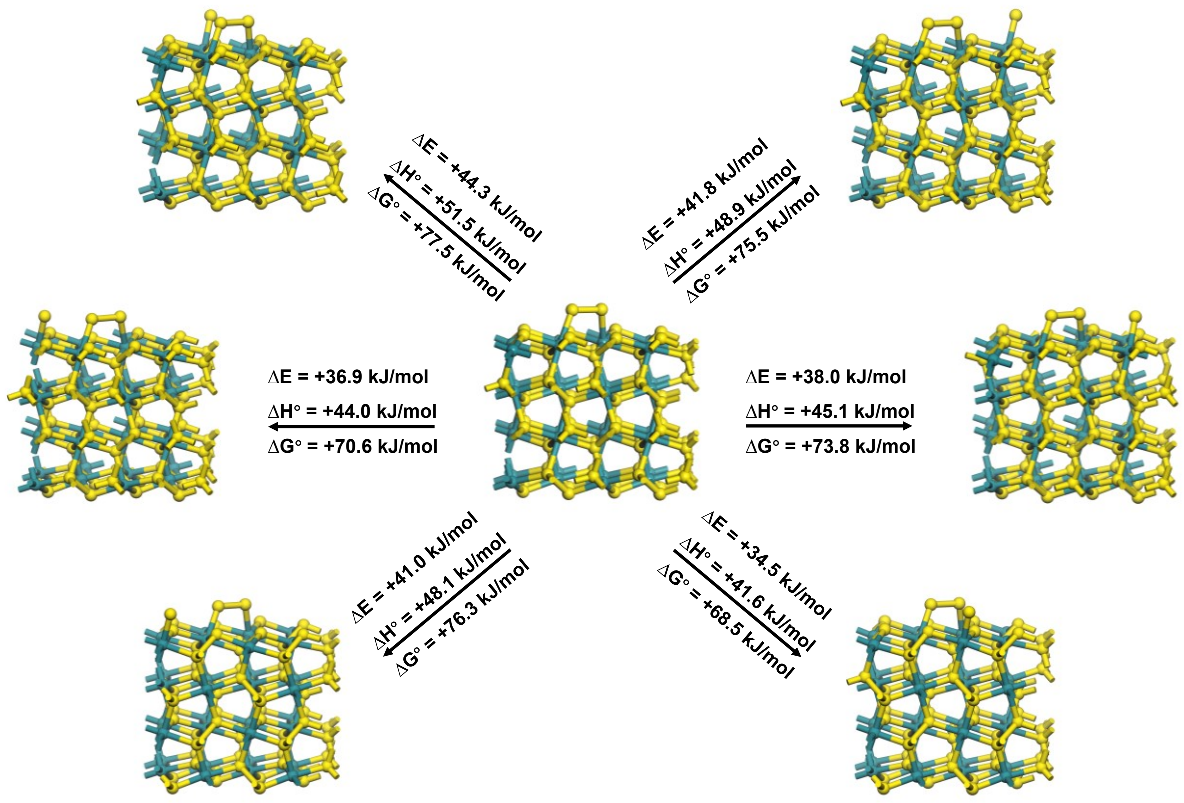

Figure S24. DFT calculations on the energy change of H_2S decomposition to H_2 and S* over RuS_2(100)_S*-S* surface. All the six Ru sites (*) around the S dimer were considered.

As shown above, S trimer cannot be formed after model optimization no matter which Ru site (*) around the S dimer was selected as the binding site for the third S atom. The corresponding $\Delta G^{\circ}_{rxn,900°C}$ for the formation of the new S monomer is around +73 kJ/mol, much higher than that for the formation of the first S monomer on the pristine RuS_2(100) surface (+24 kJ/mol, Figure 6a), indicating a possible repulsive interaction between the new S monomer and the S dimer.

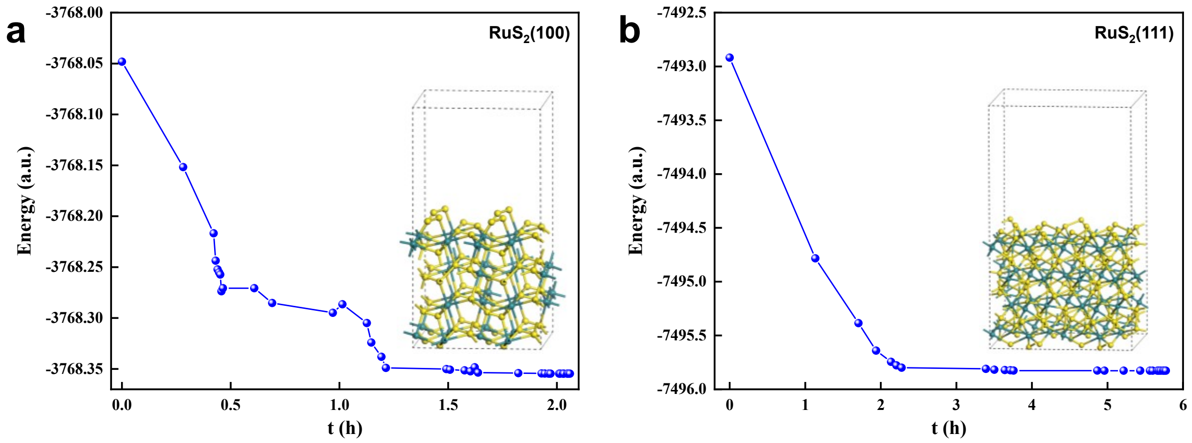

Figure S25. Model optimization for the most stable structures of S-saturated (a) $RuS_2(100)$ and (b) $RuS_2(111)$ surfaces.

The initial models were stablished by saturating all the penta-coordinated Ru sites by S atoms on both $RuS_2(100)$ and $RuS_2(111)$ surfaces. After optimization, the most stable structures on both surfaces show the formation of S dimers but no other configurations (monomer or trimer or others).

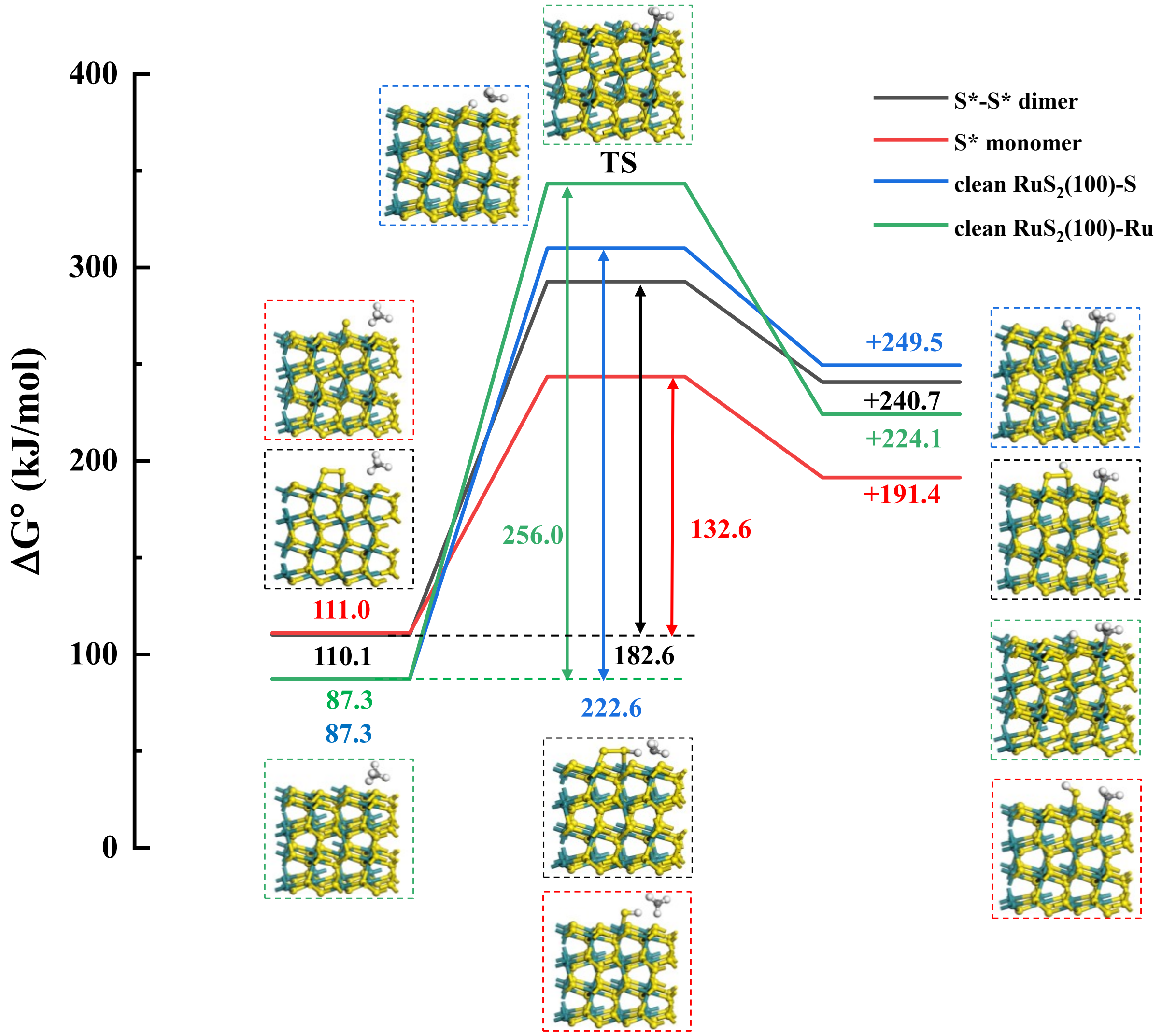

Figure S26. DFT-calculated energy barrier for the cleavage of the first C-H bond of CH_4 on different sites based on RuS_2(100) surface. The structures in the black, red, and green dotted boxes are the S*-S* dimer, S* monomer, and clean RuS_2(100) surface, respectively.

The S* monomer showed a lower energy barrier for the cleavage of the first C-H bond than the S*-S* dimer did (132.6 vs. 182.6 kJ/mol), which is reasonable considering that the singly coordinated S should be more active than the di-coordinated S. However, the coverage of S* monomer should be limited due to its relatively small $K_{H_2S,900°C}$ value (Table 2), and the S* monomer may only exist at very low partial pressures of H_2S. The cleavage of C-H bond can also occur directly on the Ru-S pair, resulting in the reduction of RuS_2 (Figure S15), even the energy barrier is as high as 222.6 or 256 kJ/mol via forming S-H or Ru-H, respectively. However, this process can only happen without the supply of H_2S (Figure S27). Thus, under our typical reaction conditions, the S*-S* dimer should be dominant on the surface of RuS_2 as jointly confirmed by the experimental and theoretical calculations (Table 2).

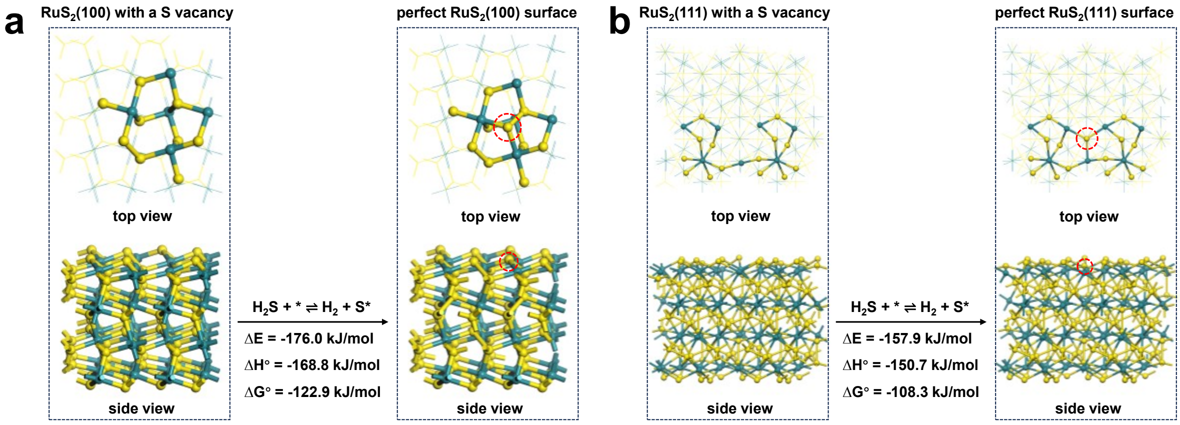

Figure S27. DFT calculations on the Gibbs free energy change of H_2S decomposition for the filling of S vacancy on (a) $RuS_2(100)$ and (b) $RuS_2(111)$ surfaces.

The Gibbs free energy changes for the filling of S-vacancy via the process of $H_2S + * \rightleftharpoons H_2 + S*$ were calculated to be very negative (-122.9 and -108.3 kJ/mol for $RuS_2(100)$ and $RuS_2(111)$, respectively), indicating that S-vacancy cannot exist under the prevailing reaction conditions. Thus, the involvement of lattice S in the catalytic cycle can also be excluded (See the detailed discussion in Situation III of Section 3 below).

Table S10. An excellent agreement in thermodynamic parameters for the main HRM reaction and H_2S decomposition at 900 °C from DFT calculations and HSC Chemistry.

Reaction	Source	$\Delta H^{\circ}_{rxn,900°C}$ (kJ/mol)	$\Delta S^{\circ}_{rxn,900°C}$ (J/mol/K)	$\Delta G^{\circ}_{rxn,900°C}$ (kJ/mol)
$CH_4 + 2H_2S \rightleftharpoons CS_2 + 4H_2$	DFT calculation	+259.1	+211.8	+10.6
	HSC Chemistry	+259.8	+213.9	+8.8
$H_2S \rightleftharpoons 0.5S_2(g) + H_2$	DFT calculation	+84.9	+48.4	+28.1
	HSC Chemistry	+90.3	+49.1	+32.7

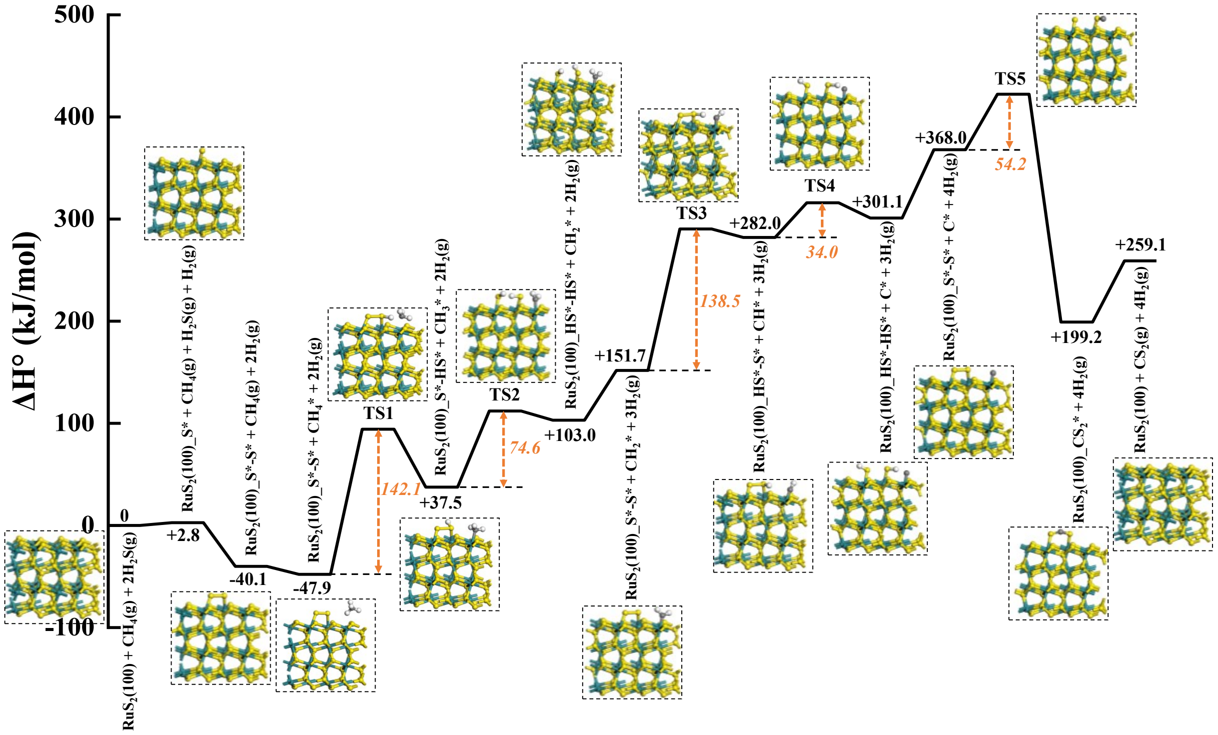

Figure S28. DFT-calculated enthalpy diagram for the HRM reaction on $RuS_2(100)$ surface. The numbers in orange color indicate the enthalpy barriers of C-H scission and CS_2 formation steps.

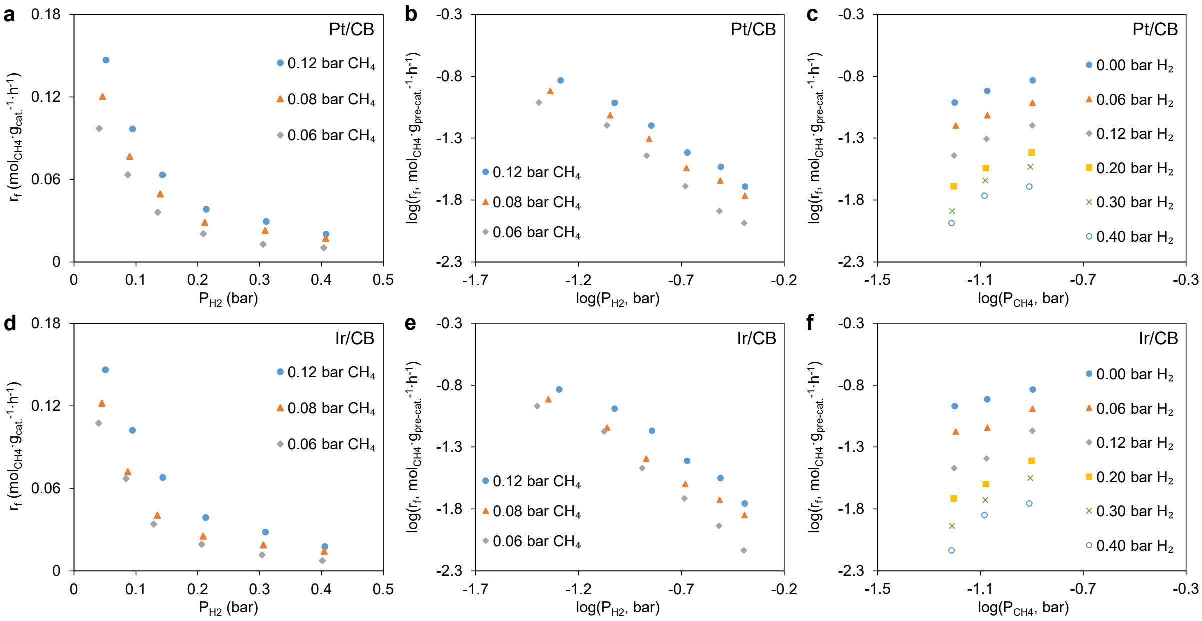

Figure S29. Influence of co-feeding H_2 on the forward rate of CH_4 conversion in HRM over (a) 5 wt% Pt/CB and (d) 5 wt% Ir/CB and the corresponding reaction orders in (b,e) H_2 and (c,f) CH_4 obtained from the H_2 co-feeding experiments. Pretreatment conditions: 20 mg of catalyst diluted with 100 mg of quartz sand, 20 mL/min of 10% H_2S in H_2 (1 bar), 900 °C, 20 min. Reaction conditions: 0-0.4 bar H_2, 0.06-0.12 bar CH_4 and 0.24 bar H_2S in He (1 bar), 150 $L \cdot g_{cat.}^{-1} \cdot h^{-1}$, 900 °C. The partial pressure of H_2 plotted here is the average pressure of the inlet (co-fed) and outlet (generated + co-fed) pressures of H_2.

Table S11. Reaction orders in CH_4 over 5 wt% Pt/CB and Ir/CB under different partial pressures of co-fed H_2 (0-0.4 bar) obtained from Figure S29.

H_2 (bar)	0	0.06	0.12	0.20	0.30	0.40
Pt/CB	0.58	0.61	0.79	0.88	1.14	0.94
Ir/CB,	0.44	0.63	1.00	1.00	1.25	1.20

As the catalytic stabilities and Pt/CB and Ir/CB are not quite good (Figure 1a), the data quality of the kinetic study shown in Figure S299 is not as nice as that for Ru/CB shown previously. Nevertheless, we can still see the same trends that the co-fed H_2 can strongly inhibit the methane conversion rate for both Pt/CB and Ir/CB (Figure S29a and S299d) and the reaction orders in CH_4 increase with increasing the partial pressure of co-fed H_2 until the extremum around 1 (Table S11). Notably, the bulk phase of Pt should be PtS under the reaction conditions, while Ir is very different that should be still in the metallic state (Figure S9).

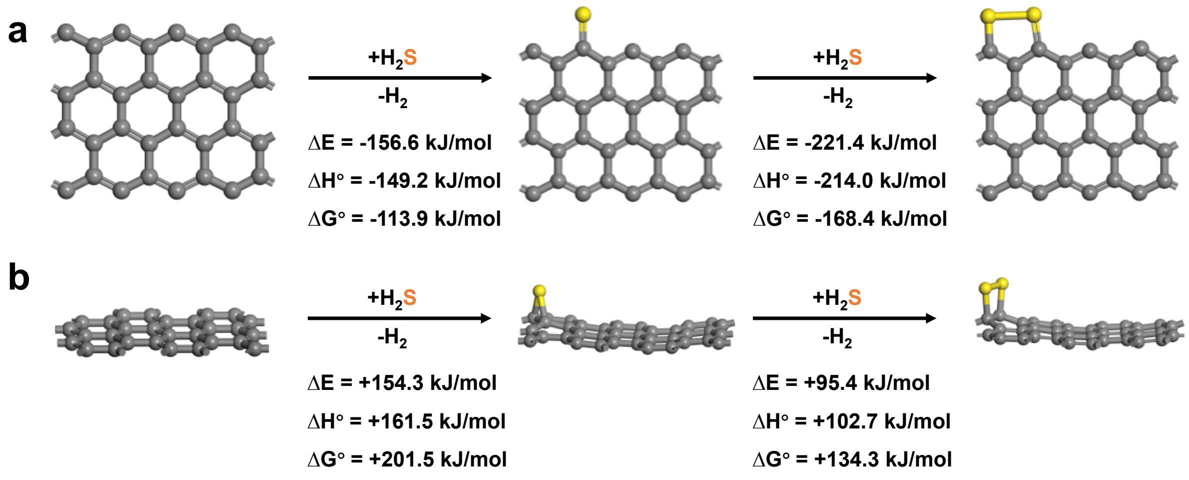

Figure S30. DFT calculations on the energy change of H_2S decomposition to H_2 and S* over (a) edge site and (b) in-plane site of graphene layer.

The energy changes of H_2S decomposition were calculated by DFT on two extreme sites of graphene layer, edge site and in-plane site. On the graphene edge site (Figure S30a), the Gibbs free energy changes for the successive decomposition of H_2S to S* monomer and S*-S* dimer were calculated to be -113.9 and -168.4 kJ/mol, respectively. Thus, the $\Delta G^{\circ}_{rxn,900°C}$ for the equilibrium of H_2S + 1/2*-* ⇌ H_2 + 1/2S*-S* is -141.2 kJ/mol, lower than that for the equilibrium of H_2S + * ⇌ H_2 + S* (-113.9 kJ/mol). On the graphene in-plane site (Figure S30b), the Gibbs free energy changes for the successive decomposition of H_2S to S* monomer and S*-S* dimer were calculated to be +201.5 and +134.3 kJ/mol, respectively. Thus, the $\Delta G^{\circ}_{rxn,900°C}$ for the equilibrium of H_2S + 1/2*-* ⇌ H_2 + 1/2S*-S* is +167.9 kJ/mol, also lower than that for the equilibrium of H_2S + * ⇌ H_2 + S* (+201.5 kJ/mol). The same trend on both sites indicates that the S*-S* dimer is thermodynamically more stable than the S* monomer, though both sites should not be the true host for active site due to either the very negative or the very negative $\Delta G^{\circ}_{rxn,900°C}$ values.

Section S3. Derivation of rate equations based on experimental findings

H-D exchange experiments demonstrate that H_2 and H_2S adsorption-desorption steps are quasi-equilibrated on the time scale of HRM catalysis (Figure 3d). Thus, we have

$$H_2S \ + * \overset{K_{H_2S}}{\Longleftrightarrow} H_2 \ + \ S * \qquad K_{H_2S} = \frac{[S*][H_2]}{[*][H_2S]}$$

$$H * \ + H * \overset{K_{H2}}{\Longleftrightarrow} H_2 \ + \ 2 * \qquad K_{H_2} = \frac{[H_2][*]^2}{[H*]^2}$$

It should be recognized that at these high temperatures, any of the H species (HS* or H*) should be present at very low concentrations at the surface, that is, they recombine and desorb as H_2 once they form. Besides the established quasi-equilibrated nature of H_2S dissociation steps and H recombination steps, the combination of C and S species may be reasonably assumed to be irreversible based on the result of CS_2 co-feeding experiments. Moreover, our isotope studies also furnished important information about the reversibility of C-H bond dissociation steps. Clear evidence (CH_xD_{4-x} isotopomer distribution, x = 0-4) showed that at least the first C-H bond scission is reversible, but not all C-H scission steps are quasi-equilibrated at the prevalent conditions used in this work. To derive rate equations, two extreme cases were considered upon the reversibility of C-H bond scission, one is to assume that the first C-H bond scission is the only reversible step, the other is to assume that the scission of the other three C-H bonds are quasi-equilibrated. The other cases in between will increase the complexity of the derived rate equation but not change the general form.

In the following, we present three situations of reaction mechanisms. The first two are based on S*-assisted activation of C-H bonds in methane that differ in the species (and its binding site) formed upon the first C-H bond scission, which is thought to form either H_3C* or H_3CS*. The third one is the so-called Mars-van-Krevelen (MvK) mechanism, that is, methane is activated by the lattice S-Ru pair.

Situation I: H_3C* is assumed to be formed after the first C-H bond scission assisted by S∗.

Subcase 1: The first C-H bond scission is assumed to be the only reversible step during the decomposition of methane.

$$CH_4 + * \ + S * \underset{k_{-1}}{\overset{k_1}{\rightleftharpoons}} CH_3 * \ + HS *$$

$$CH_3 * \ + S * \overset{k_2}{\rightarrow} CH_2 * \ + HS *$$

$$CH_2 * \ + S * \overset{k_3}{\rightarrow} CH * \ + HS *$$

$CH* + S* \overset{k_4}{\rightarrow} C* + HS*$

$C* + S* \overset{k_5}{\rightarrow} SC* + *$

$SC* + S* \overset{k_6}{\rightarrow} CS_2 + 2*$

Then, the rate can be expressed as

$rate = k_1[CH_4][*][S*] - k_{-1}[CH_3*][HS*] = k_2[CH_3*][S*] = k_3[CH_2*][S*] = k_4[CH*][S*] = k_5[C*][S*] = k_6[SC*][S*]$

To facilitate the derivation, the elemental steps of H_2S decomposition are appended as below:

$H_2S + * + * \overset{K_7}{\Leftrightarrow} HS* + H*$ $\quad K_7 = \frac{[HS*][H*]}{[H_2S][*]^2}$

$HS* + * \overset{K_8}{\Leftrightarrow} S* + H*$ $\quad K_8 = \frac{[S*][H*]}{[HS*][*]}$

Then, correlations between $[HS*]$, $[S*]$ and $[*]$ can be obtained as follows:

$$K_8 {K_{H_2}}^{0.5} = \frac{[S*][H_2]^{0.5}}{[HS*]}$$

$$K_{H_2S} = K_7 K_8 K_{H_2} = \frac{[S*][H_2]}{[H_2S][*]}$$

After taking them into the above rate equation, a correlation between $[CH_3*]$ and $[*]$ can be obtained:

$$[CH_3*] = \frac{k_1[CH_4][*][S*]}{k_2[S*]+k_{-1}[HS*]} = \frac{k_1[CH_4][*][S*]}{k_2[S*]+k_{-1}[S*]\frac{[H_2]^{0.5}}{K_8{K_{H_2}}^{0.5}}} = \frac{k_1[CH_4]}{k_2+\frac{k_{-1}[H_2]^{0.5}}{K_8{K_{H_2}}^{0.5}}}[*]$$

Applying the site balance equation for total exposed metal cation sites,

$$[CH_3*] + [CH_2*] + [CH*] + [C*] + [SC*] + [S*] + [*] = [*]_{total}$$

$$\left\{\left(1 + \frac{k_2}{k_3} + \frac{k_2}{k_4} + \frac{k_2}{k_5} + \frac{k_2}{k_6}\right) \times \frac{k_1[CH_4]}{k_2+\frac{k_{-1}[H_2]^{0.5}}{K_8{K_{H_2}}^{0.5}}} + K_{H_2S}\frac{[H_2S]}{[H_2]} + 1\right\}[*] = [*]_{total}$$

The final equation can be obtained as below:

$$r_1 = k_2[CH_3*][S*] = \frac{k_1k_2[CH_4][*][S*]}{k_2+\frac{k_{-1}[H_2]^{0.5}}{K_8{K_{H_2}}^{0.5}}} = \frac{k_1k_2[CH_4]K_{H_2S}\frac{[H_2S]}{[H_2]}[*]^2}{k_2+\frac{k_{-1}[H_2]^{0.5}}{K_8{K_{H_2}}^{0.5}}}$$

$$r_1 = \frac{k_1k_2[CH_4]K_{H_2S}\frac{[H_2S]}{[H_2]}}{\left(k_2+\frac{k_{-1}[H_2]^{0.5}}{K_8{K_{H_2}}^{0.5}}\right)\left\{\left(1+\frac{k_2}{k_3}+\frac{k_2}{k_4}+\frac{k_2}{k_5}+\frac{k_2}{k_6}\right)\times\frac{k_1[CH_4]}{k_2+\frac{k_{-1}[H_2]^{0.5}}{K_8{K_{H_2}}^{0.5}}}+K_{H_2S}\frac{[H_2S]}{[H_2]}+1\right\}^2}$$

At conditions where the reaction order in methane becomes 1 (e.g., 0.2-0.6 bar H_2 pressure), the rate equation can be simplified to:

$$r_1 = \frac{k_1 k_2 [CH_4] K_{H_2S} \frac{[H_2S]}{[H_2]}}{\left(k_2 + \frac{k_{-1}[H_2]^{0.5}}{K_8 K_{H_2}{}^{0.5}}\right)\left(K_{H_2S}\frac{[H_2S]}{[H_2]}+1\right)^2}$$

Subcase 2: All the steps of C-H bond scission are assumed to be reversible and the last three are assumed to reach quasi-equilibration.

$$CH_4 + * + S* \underset{k_{-1}}{\overset{k_1}{\rightleftharpoons}} CH_3* + HS*$$

$$CH_3* + S* \overset{K_2}{\Leftrightarrow} CH_2* + HS* \qquad K_2 = \frac{[CH_2*][HS*]}{[CH_3*][S*]}$$

$$CH_2* + S* \overset{K_3}{\Leftrightarrow} CH* + HS* \qquad K_3 = \frac{[CH*][HS*]}{[CH_2*][S*]}$$

$$CH* + S* \overset{K_4}{\Leftrightarrow} C* + HS* \qquad K_4 = \frac{[C*][HS*]}{[CH*][S*]}$$

$$C* + S* \overset{k_5}{\rightarrow} SC* + *$$

$$SC* + S* \overset{k_6}{\rightarrow} CS_2 + 2*$$

Then, the rate can be expressed as

$$rate = k_1[CH_4][*][S*] - k_{-1}[CH_3*][HS*] = k_5[C*][S*] = k_6[SC*][S*]$$

To facilitate the derivation, the elemental steps of H_2S decomposition are appended as below:

$$H_2S + * + * \overset{K_7}{\Leftrightarrow} HS* + H* \qquad K_7 = \frac{[HS*][H*]}{[H_2S][*]^2}$$

$$HS* + * \overset{K_8}{\Leftrightarrow} S* + H* \qquad K_8 = \frac{[S*][H*]}{[HS*][*]}$$

Then, correlations between $[HS*]$, $[S*]$ and $[*]$ can be obtained as follows:

$$K_8 K_{H_2}{}^{0.5} = \frac{[S*][H_2]^{0.5}}{[HS*]}$$

$$K_{H_2S} = K_7 K_8 K_{H_2} = \frac{[S*][H_2]}{[H_2S][*]}$$

After taking them into the above rate equation, a correlation between $[CH_3*]$, $[CH_2*]$, $[CH*]$, $[C*]$ and $[*]$ can be obtained:

$$[CH*] = \frac{[C*][HS*]}{K_4[S*]} = \frac{[H_2]^{0.5}}{K_4 K_8 K_{H_2}{}^{0.5}}[C*]$$

$$[CH_2*] = \frac{[CH*][HS*]}{K_3[S*]} = \frac{[H_2]^{0.5}}{K_3 K_8 K_{H_2}{}^{0.5}}[CH*] = \frac{[H_2]}{K_3 K_4 K_8{}^2 K_{H_2}}[C*]$$

$$[CH_3*] = \frac{[CH_2*][HS*]}{K_2[S*]} = \frac{[H_2]^{0.5}}{K_2 K_8 K_{H_2}{}^{0.5}}[CH_2*] = \frac{[H_2]^{1.5}}{K_2 K_3 K_4 K_8{}^3 K_{H_2}{}^{1.5}}[C*]$$

$$[C*] = \frac{k_1[CH_4][*][S*]}{k_5[S*] + k_{-1}\frac{[H_2]^{1.5}}{K_2K_3K_4K_8{}^3K_{H_2}{}^{1.5}}[HS*]} = \frac{k_1[CH_4][*][S*]}{k_5[S*] + k_{-1}\frac{[H_2]^{1.5}}{K_2K_3K_4K_8{}^3K_{H_2}{}^{1.5}}[S*]\frac{[H_2]^{0.5}}{K_8K_{H_2}{}^{0.5}}} = \frac{k_1[CH_4]}{k_5 + \frac{k_{-1}[H_2]^2}{K_2K_3K_4K_8{}^4K_{H_2}{}^2}}[*]$$

Applying the site balance equation for total exposed metal cation sites,

$[CH_3 *] + [CH_2 *] + [CH *] + [C *] + [SC *] + [S *] + [*] = [*]_{total}$

$$\left\{\left(\frac{[H_2]^{1.5}}{K_2K_3K_4K_8{}^3K_{H_2}{}^{1.5}} + \frac{[H_2]}{K_3K_4K_8{}^2K_{H_2}} + \frac{[H_2]^{0.5}}{K_4K_8K_{H_2}{}^{0.5}} + 1 + \frac{k_5}{k_6}\right) \times \frac{k_1[CH_4]}{k_5 + \frac{k_{-1}[H_2]^2}{K_2K_3K_4K_8{}^4K_{H_2}{}^2}} + K_{H_2S}\frac{[H_2S]}{[H_2]} + 1\right\}[*] = [*]_{total}$$

The final equation can be obtained as below:

$$r_1' = k_5[C *][S *] = \frac{k_5k_1[CH_4][*][S*]}{k_5 + \frac{k_{-1}[H_2]^2}{K_2K_3K_4K_8{}^4K_{H_2}{}^2}} = \frac{k_5k_1[CH_4]K_{H_2S}\frac{[H_2S]}{[H_2]}[*]^2}{k_5 + \frac{k_{-1}[H_2]^2}{K_2K_3K_4K_8{}^4K_{H_2}{}^2}}$$

$$r_1' = \frac{k_1k_5[CH_4]K_{H_2S}\frac{[H_2S]}{[H_2]}}{\left(k_5 + \frac{k_{-1}[H_2]^2}{K_2K_3K_4K_8{}^4K_{H_2}{}^2}\right)\left\{\left(\frac{[H_2]^{1.5}}{K_2K_3K_4K_8{}^3K_{H_2}{}^{1.5}} + \frac{[H_2]}{K_3K_4K_8{}^2K_{H_2}} + \frac{[H_2]^{0.5}}{K_4K_8K_{H_2}{}^{0.5}} + 1 + \frac{k_5}{k_6}\right) \times \frac{k_1[CH_4]}{k_5 + \frac{k_{-1}[H_2]^2}{K_2K_3K_4K_8{}^4K_{H_2}{}^2}} + K_{H_2S}\frac{[H_2S]}{[H_2]} + 1\right\}^2}$$

At conditions where the reaction order in methane becomes 1 (e.g., 0.2-0.6 bar H_2 pressure), the rate equation can be simplified to:

$$r_1' = \frac{k_1k_5[CH_4]K_{H_2S}\frac{[H_2S]}{[H_2]}}{\left(k_5 + \frac{k_{-1}[H_2]^2}{K_2K_3K_4K_8{}^4K_{H_2}{}^2}\right)\left(K_{H_2S}\frac{[H_2S]}{[H_2]} + 1\right)^2}$$

Situation II: H_3CS* is assumed to be formed after the first C-H bond scission assisted by S∗.

Subcase 1: The first C-H bond scission is assumed to be the only reversible step during the decomposition of methane.

$$CH_4 + S* + S* \underset{k_{-1}}{\overset{k_1}{\rightleftharpoons}} CH_3S* + HS*$$

$$CH_3S* + S* \overset{k_2}{\rightarrow} CH_2S* + HS*$$

$$CH_2S* + S* \overset{k_3}{\rightarrow} CHS* + HS*$$

$$CHS* + S* \overset{k_4}{\rightarrow} CS* + HS*$$

$$CS* + S* \overset{k_5}{\rightarrow} CS_2 + 2*$$

Then, the rate can be expressed as:

$rate = k_1[CH_4][S *][S *] - k_{-1}[CH_3S *][HS *] = k_2[CH_3S *][S *] = k_3[CH_2S *][S *] = k_4[CHS *][S *] = k_5[CS *][S *]$

To facilitate the derivation, the elemental steps of H_2S decomposition are appended as below:

$$H_2S + * + * \overset{K_6}{\Leftrightarrow} HS* + H* \qquad K_6 = \frac{[HS*][H*]}{[H_2S][*]^2}$$

$$HS* + * \overset{K_7}{\Leftrightarrow} S* + H* \qquad K_7 = \frac{[S*][H*]}{[HS*][*]}$$

From the above equations, correlations between $[HS*]$, $[S*]$ and $[*]$ can be obtained as follows:

$$K_7 K_{H_2}{}^{0.5} = \frac{[S*][H_2]^{0.5}}{[HS*]}$$

$$K_{H_2S} = K_6 K_7 K_{H_2} = \frac{[S*][H_2]}{[H_2S][*]}$$

After taking them into the above rate equation, a correlation between $[CH_3S*]$ and $[S*]$ can be obtained:

$$[CH_3S*] = \frac{k_1[CH_4][S*][S*]}{k_2[S*]+k_{-1}[HS*]} = \frac{k_1[CH_4][S*][S*]}{k_2[S*]+k_{-1}[S*]\frac{[H_2]^{0.5}}{K_7K_{H_2}{}^{0.5}}} = \frac{k_1[CH_4]}{k_2+\frac{k_{-1}[H_2]^{0.5}}{K_7K_{H_2}{}^{0.5}}}[S*]$$

Applying the site balance equation for the total exposed metal cation sites:

$$[CH_3S*] + [CH_2S*] + [CHS*] + [CS*] + [S*] + [*] = [*]_{total}$$

$$\left\{\left[\left(1+\frac{k_2}{k_3}+\frac{k_2}{k_4}+\frac{k_2}{k_5}\right)\times\frac{k_1[CH_4]}{k_2+\frac{k_{-1}[H_2]^{0.5}}{K_7K_{H_2}{}^{0.5}}}+1\right]\times K_{H_2S}\frac{[H_2S]}{[H_2]}+1\right\}[*] = [*]_{total}$$

The final equation can be obtained as below:

$$r_2 = k_2[CH_3S*][S*] = \frac{k_1k_2[CH_4][S*][S*]}{k_2+\frac{k_{-1}[H_2]^{0.5}}{K_7K_{H_2}{}^{0.5}}} = \frac{k_1k_2[CH_4]}{k_2+\frac{k_{-1}[H_2]^{0.5}}{K_7K_{H_2}{}^{0.5}}}\times\left(K_{H_2S}\frac{[H_2S]}{[H_2]}[*]\right)^2$$

$$r_2 = \frac{k_1k_2[CH_4]\left(K_{H_2S}\frac{[H_2S]}{[H_2]}\right)^2}{\left(k_2+\frac{k_{-1}[H_2]^{0.5}}{K_7K_{H_2}{}^{0.5}}\right)\left\{\left[\left(1+\frac{k_2}{k_3}+\frac{k_2}{k_4}+\frac{k_2}{k_5}\right)\times\frac{k_1[CH_4]}{k_2+\frac{k_{-1}[H_2]^{0.5}}{K_7K_{H_2}{}^{0.5}}}+1\right]\times K_{H_2S}\frac{[H_2S]}{[H_2]}+1\right\}^2}$$

At conditions where the reaction order in methane becomes 1 (e.g., 0.2-0.6 bar H_2 pressure), the rate equation can be simplified to:

$$r_2 = \frac{k_1k_2[CH_4]\left(K_{H_2S}\frac{[H_2S]}{[H_2]}\right)^2}{\left(k_2+\frac{k_{-1}[H_2]^{0.5}}{K_7K_{H_2}{}^{0.5}}\right)\left(K_{H_2S}\frac{[H_2S]}{[H_2]}+1\right)^2}$$

Subcase 2: All the steps of C-H bond scission are assumed to be reversible and the last three are assumed to reach quasi-equilibration.

$$CH_4 + S* + S* \underset{k_{-1}}{\overset{k_1}{\rightleftharpoons}} CH_3S* + HS*$$

$$CH_3S* + S* \overset{K_2}{\Leftrightarrow} CH_2S* + HS* \qquad K_2 = \frac{[CH_2S*][HS*]}{[CH_3S*][S*]}$$

$$CH_2S* + S* \overset{K_3}{\Leftrightarrow} CHS* + HS* \qquad K_3 = \frac{[CHS*][HS*]}{[CH_2S*][S*]}$$

$$CHS* + S* \overset{K_4}{\Leftrightarrow} CS* + HS* \qquad K_4 = \frac{[CS*][HS*]}{[CHS*][S*]}$$

$$CS* + S* \overset{k_5}{\rightarrow} CS_2 + 2*$$

Then, the rate can be expressed as:

$$rate = k_1[CH_4][S*][S*] - k_{-1}[CH_3S*][HS*] = k_5[CS*][S*]$$

To facilitate the derivation, the elemental steps of H_2S decomposition are appended as below:

$$H_2S + * + * \overset{K_6}{\Leftrightarrow} HS* + H* \qquad K_6 = \frac{[HS*][H*]}{[H_2S][*]^2}$$

$$HS* + * \overset{K_7}{\Leftrightarrow} S* + H* \qquad K_7 = \frac{[S*][H*]}{[HS*][*]}$$

From the above equations, correlations between $[HS*]$, $[S*]$ and $[*]$ can be obtained as follows:

$$K_7 {K_{H_2}}^{0.5} = \frac{[S*][H_2]^{0.5}}{[HS*]}$$

$$K_{H_2S} = K_6 K_7 K_{H_2} = \frac{[S*][H_2]}{[H_2S][*]}$$

After taking them into the above rate equation, a correlation between $[CH_3S*]$, $[CH_2S*]$, $[CHS*]$, $[CS*]$, and $[S*]$ can be obtained:

$$[CHS*] = \frac{[CS*][HS*]}{K_4[S*]} = \frac{[H_2]^{0.5}}{K_4K_7{K_{H_2}}^{0.5}}[CS*]$$

$$[CH_2S*] = \frac{[CHS*][HS*]}{K_3[S*]} = \frac{[H_2]^{0.5}}{K_3K_7{K_{H_2}}^{0.5}}[CHS*] = \frac{[H_2]}{K_3K_4{K_7}^2K_{H_2}}[CS*]$$

$$[CH_3S*] = \frac{[CH_2S*][HS*]}{K_2[S*]} = \frac{[H_2]^{0.5}}{K_2K_7{K_{H_2}}^{0.5}}[CH_2S*] = \frac{[H_2]^{1.5}}{K_2K_3K_4{K_7}^3{K_{H_2}}^{1.5}}[CS*]$$

$$[CS*] = \frac{k_1[CH_4][S*][S*]}{k_5[S*] + k_{-1}\frac{[H_2]^{1.5}}{K_2K_3K_4{K_7}^3{K_{H_2}}^{1.5}}[HS*]} = \frac{k_1[CH_4][S*][S*]}{k_5[S*] + k_{-1}\frac{[H_2]^{1.5}}{K_2K_3K_4{K_7}^3{K_{H_2}}^{1.5}}[S*]\frac{[H_2]^{0.5}}{K_7{K_{H_2}}^{0.5}}} = \frac{k_1[CH_4]}{k_5 + \frac{k_{-1}[H_2]^2}{K_2K_3K_4{K_7}^4{K_{H_2}}^2}}[S*]$$

Applying the site balance equation for the total exposed metal cation sites:

$$[CH_3S*] + [CH_2S*] + [CHS*] + [CS*] + [S*] + [*] = [*]_{total}$$

$$\left\{\left[\left(\frac{[H_2]^{1.5}}{K_2K_3K_4{K_7}^3{K_{H_2}}^{1.5}} + \frac{[H_2]}{K_3K_4{K_7}^2K_{H_2}} + \frac{[H_2]^{0.5}}{K_4K_7{K_{H_2}}^{0.5}} + 1\right) \times \frac{k_1[CH_4]}{k_5 + \frac{k_{-1}[H_2]^2}{K_2K_3K_4{K_7}^4{K_{H_2}}^2}} + 1\right] \times K_{H_2S}\frac{[H_2S]}{[H_2]} + 1\right\}[*] = [*]_{total}$$

The final equation can be obtained as below:

$$r_2' = k_5[CS*][S*] = \frac{k_5k_1[CH_4][S*][S*]}{k_5 + \frac{k_{-1}[H_2]^2}{K_2K_3K_4{K_7}^4{K_{H_2}}^2}} = \frac{k_5k_1[CH_4]}{k_5 + \frac{k_{-1}[H_2]^2}{K_2K_3K_4{K_7}^4{K_{H_2}}^2}} \times \left(K_{H_2S}\frac{[H_2S]}{[H_2]}[*]\right)^2$$

$$r_2' = \frac{k_1k_5[CH_4]\left(K_{H_2S}\frac{[H_2S]}{[H_2]}\right)^2}{\left(k_5 + \frac{k_{-1}[H_2]^2}{K_2K_3K_4{K_7}^4{K_{H_2}}^2}\right)\left\{\left[\left(\frac{[H_2]^{1.5}}{K_2K_3K_4{K_7}^3{K_{H_2}}^{1.5}} + \frac{[H_2]}{K_3K_4{K_7}^2K_{H_2}} + \frac{[H_2]^{0.5}}{K_4K_7{K_{H_2}}^{0.5}} + 1\right) \times \frac{k_1[CH_4]}{k_5 + \frac{k_{-1}[H_2]^2}{K_2K_3K_4{K_7}^4{K_{H_2}}^2}} + 1\right] \times K_{H_2S}\frac{[H_2S]}{[H_2]} + 1\right\}^2}$$

At conditions where the reaction order in methane becomes 1 (e.g., 0.2-0.6 bar H_2 pressure), the rate equation can be simplified to:

$$r_2' = \frac{k_1 k_5 [CH_4]\left(K_{H_2S}\frac{[H_2S]}{[H_2]}\right)^2}{\left(k_5 + \frac{k_{-1}[H_2]^2}{K_2 K_3 K_4 K_7{}^4 K_{H_2}{}^2}\right)\left(K_{H_2S}\frac{[H_2S]}{[H_2]}+1\right)^2}$$

Situation III: CH_4 is assumed to be activated by a metal cation (∗) – lattice sulfur anion (#) site pair, with CH_3 fragment bound on ∗ and detached H transiently accepted by permanent lattice sulfur (#) but not by S∗, and CS_2 is formed with two adjacent lattice sulfur (#).

$$CH_4 + * + \# \underset{k_{-1}}{\overset{k_1}{\rightleftharpoons}} CH_3* + H\#$$

$$CH_3* + \# \overset{k_2}{\rightarrow} CH_2* + H\#$$

$$CH_2* + \# \overset{k_3}{\rightarrow} CH* + H\#$$

$$CH* + \# \overset{k_4}{\rightarrow} C* + H\#$$

$$C* + \# \overset{k_5}{\rightarrow} SC* + \square$$

$$SC* + \# \overset{k_6}{\rightarrow} CS_2 + * + \square$$

Then, the rate can be expressed as

$$rate = k_1[CH_4][*][\#] - k_{-1}[CH_3*][H\#] = k_2[CH_3*][\#] = k_3[CH_2*][\#] =$$
$$k_4[CH*][\#] = k_5[C*][\#] = k_6[SC*][\#]$$

After rearranging, a correlation between $[CH_3*]$ and $[*]$ can be obtained as follows.

$$[CH_3*] = \frac{k_1[CH_4][*][\#]}{k_2[\#]+k_{-1}[H\#]} = \frac{k_1[CH_4][*][\#]}{k_2[\#]+k_{-1}[\#]\frac{[H_2]^{0.5}}{K_{H_2}{}^{0.5}}} = \frac{k_1[CH_4]}{k_2+\frac{k_{-1}[H_2]^{0.5}}{K_{H_2}{}^{0.5}}}[*]$$

There are two sets of site balance for this situation, one applying to total exposed metal cation sites:

$$[CH_3*] + [CH_2*] + [CH*] + [C*] + [SC*] + [S*] + [*] = [*]_{total}$$

$$\left\{\left(1+\frac{k_2}{k_3}+\frac{k_2}{k_4}+\frac{k_2}{k_5}+\frac{k_2}{k_6}\right)\times\frac{k_1[CH_4]}{k_2+\frac{k_{-1}[H_2]^{0.5}}{K_{H_2}{}^{0.5}}} + K_{H_2S}\frac{[H_2S]}{[H_2]} + 1\right\}[*] = [*]_{total}$$

The other applying to lattice sulfur anion sites:

$$[\#] + \square = [\#]_{total}$$

which will be determined by the decomposition of H_2S.

$$[\#] = \frac{K'_{H_2S}\frac{[H_2S]}{[H_2]}}{1+K'_{H_2S}\frac{[H_2S]}{[H_2]}}[\#]_{total}$$

The final equation can be obtained as below.

$$r_3 = k_2[CH_3*][\#] = \frac{k_1 k_2 [CH_4][*][\#]}{k_2 + \frac{k_{-1}[H_2]^{0.5}}{K_{H_2}{}^{0.5}}}$$

$$r_3 = \frac{k_1 k_2 [CH_4] K'_{H_2S} \frac{[H_2S]}{[H_2]}}{\left(k_2 + \frac{k_{-1}[H_2]^{0.5}}{K_{H_2}{}^{0.5}}\right)\left\{\left(1+\frac{k_2}{k_3}+\frac{k_2}{k_4}+\frac{k_2}{k_5}+\frac{k_2}{k_6}\right)\times\frac{k_1[CH_4]}{k_2+\frac{k_{-1}[H_2]^{0.5}}{K_{H_2}{}^{0.5}}}+K_{H_2S}\frac{[H_2S]}{[H_2]}+1\right\}\left(1+K'_{H_2S}\frac{[H_2S]}{[H_2]}\right)}$$

For the filling of S-vacancies (□) on RuS_2, the equilibrium constant K'_{H_2S} was calculated to be around 10^5 by DFT, which is very large (the corresponding $\Delta G^{\circ}_{rxn,900°C}$ is around -115 kJ/mol, Figure S27). Thus, the rate equation can be simplified to:

$$r_3 = \frac{k_1 k_2 [CH_4]}{\left(k_2 + \frac{k_{-1}[H_2]^{0.5}}{K_{H_2}{}^{0.5}}\right)\left\{\left(1+\frac{k_2}{k_3}+\frac{k_2}{k_4}+\frac{k_2}{k_5}+\frac{k_2}{k_6}\right)\times\frac{k_1[CH_4]}{k_2+\frac{k_{-1}[H_2]^{0.5}}{K_{H_2}{}^{0.5}}}+K_{H_2S}\frac{[H_2S]}{[H_2]}+1\right\}}$$

From the above rate equation, one can see that the reaction order in H_2S cannot be positive, which is in conflict with our experimental data. Thus, the MvK mechanism is unlikely.
